# Implementation and sustainability factors of two early-stage breast cancer conversation aids in diverse practices

**DOI:** 10.1186/s13012-021-01115-1

**Published:** 2021-05-10

**Authors:** Danielle Schubbe, Renata W. Yen, Catherine H. Saunders, Glyn Elwyn, Rachel C. Forcino, A. James O’Malley, Mary C. Politi, Julie Margenthaler, Robert J. Volk, Karen Sepucha, Elissa Ozanne, Sanja Percac-Lima, Ann Bradley, Courtney Goodwin, Maria van den Muijsenbergh, Johanna W. M. Aarts, Peter Scalia, Marie-Anne Durand

**Affiliations:** 1 The Dartmouth Institute for Health Policy and Clinical Practice, Dartmouth College, Lebanon, NH USA; 2 Dartmouth-Hitchcock Medical Center, Lebanon, NH USA; 3 Department of Surgery, Washington University School of Medicine, St. Louis, MO USA; 4 Division of Cancer Prevention & Population Sciences, The University of Texas MD Anderson Cancer Center, Houston, TX USA; 5 Division of General Internal Medicine, Massachusetts General Hospital, Boston, MA USA; 6 University of Utah, Salt Lake City, UT USA; 7 Massachusetts General Hospital’s Chelsea Healthcare Center, Chelsea, MA USA; 8 RadboudUMC, Nijmegen, The Netherlands; 9 AmsterdamUMC, Amsterdam, The Netherlands; 10 UMR 1295, CERPOP, Université de Toulouse, Inserm, Université Toulouse III Paul Sabatier, 37 Allées Jules Guesde, 31000 Toulouse, France; 11 Unisanté, Centre universitaire de médecine générale et santé publique, Rue du Bugnon 44, CH-1011 Lausanne, Switzerland

**Keywords:** Encounter patient decision aid, Conversation aid, Decision aid, Encounter decision aid, Health communication, Shared decision-making, Implementation, Breast cancer, Normalization Process Theory, Qualitative research

## Abstract

**Background:**

Conversation aids can facilitate shared decision-making and improve patient-centered outcomes. However, few examples exist of sustained use of conversation aids in routine care due to numerous barriers at clinical and organizational levels. We explored factors that will promote the sustained use of two early-stage breast cancer conversation aids. We examined differences in opinions between the two conversation aids and across socioeconomic strata.

**Methods:**

We nested this study within a randomized controlled trial that demonstrated the effectiveness of two early-stage breast cancer surgery conversation aids, one text-based and one picture-based. These conversation aids facilitated more shared decision-making and improved the decision process, among other outcomes, across four health systems with socioeconomically diverse patient populations. We conducted semi-structured interviews with a purposive sample of patient participants across conversation aid assignment and socioeconomic status (SES) and collected observations and field notes. We interviewed trial surgeons and other stakeholders. Two independent coders conducted framework analysis using the NOrmalization MeAsure Development through Normalization Process Theory. We also conducted an inductive analysis. We conducted additional sub-analyses based on conversation aid assignment and patient SES.

**Results:**

We conducted 73 semi-structured interviews with 43 patients, 16 surgeons, and 14 stakeholders like nurses, cancer center directors, and electronic health record (EHR) experts. Patients and surgeons felt the conversation aids should be used in breast cancer care in the future and were open to various methods of giving and receiving the conversation aid (EHR, email, patient portal, before consultation). Patients of higher SES were more likely to note the conversation aids influenced their treatment discussion, while patients of lower SES noted more influence on their decision-making. Intervention surgeons reported using the conversation aids did not lengthen their typical consultation time. Most intervention surgeons felt using the conversation aids enhanced their usual care after using it a few times, and most patients felt it appeared part of their normal routine.

**Conclusions:**

Key factors that will guide the future sustained implementation of the conversation aids include adapting to existing clinical workflows, flexibility of use, patient characteristics, and communication preferences.

**Trial registration:**

ClinicalTrials.gov Identifier: NCT03136367, registered on May 2, 2017

**Supplementary Information:**

The online version contains supplementary material available at 10.1186/s13012-021-01115-1.

Contributions to the literature

- Both surgeons and patients, regardless of conversation aid used and socioeconomic status, recommended the early-stage breast cancer conversation aids be used in future breast cancer care.
- Tailoring the use of conversation aids to existing clinical workflows, flexibility of use, and taking into account patient characteristics and preferences, like health literacy, can facilitate sustained implementation.
- Normalization Process Theory (originally focused on health professionals’ perspectives) can also feasibly be used to analyze the patient perspective about the sustainable implementation of conversation aids into diverse practices.

## Introduction

Conversation aids help patients compare treatment options using evidence-based information to support them and their families in making a decision that is aligned with their preferences and values [1–3]. However, sustained implementation of these conversation aids in clinical care is complex [4–13]. Clinical barriers faced by clinicians can include a lack of training on the conversation aids, indifference to using them, lack of trust in the content, and possible disruption of established clinic behaviors and workflows [5]. While training is recognized by clinic staff as an important way to promote shared decision-making and the use of conversation aids, a common barrier to implementation is the lack of appropriate training on how best to use and integrate the conversation aids [4, 5, 14].

In breast cancer care, clinicians tend to feel they communicate well with their patients and are reluctant to modify aspects of their usual care [15]. Clinicians also worry that it takes too much time to use a conversation aid and engage in shared decision-making [14, 16]. Buy-in at the clinic level, led by clinical champions, can reinforce the sustainability of an implemented conversation aid [17]. Despite the multiple barriers that exist, tailoring to the individual needs of clinics can ensure better sustainability of an implemented conversation aid [18]. System-level barriers can include a lack of prioritizing implementation efforts and incentivization for clinicians and clinic staff [5–7]. An organization’s willingness to adhere to guidelines promotes the implementation of conversation aids [19].

Patients with early-stage breast cancer are faced with a preference-sensitive decision, where their treatment options (mastectomy or breast-conserving surgery with radiation) need to be deliberated, ideally using shared decision-making [20–22]. Patients of lower socioeconomic status (SES) who have early-stage breast cancer often have poorer communication with their doctors and health outcomes [23–27]. We conducted a multi-site randomized controlled trial randomizing surgeons to one of two conversation aids for early-stage breast cancer treatment options or to usual care. One conversation aid was text-based (Option Grid), and the other was picture-enhanced (Picture Option Grid). Picture Option Grid resulted in higher knowledge and improved decision process (primary outcome), lower decision regret, and higher self-reported and observed shared decision-making compared with usual care. Option Grid resulted in improved decision process (primary outcome), more coordinated care, and observed shared decision-making compared with usual care. Neither intervention impacted preference concordance (third subscale of primary outcome measure). There were no statistically significant differences between the interventions for all outcomes measured. Compared with usual care, Picture Option Grid had more impact on knowledge and quality of life among disadvantaged patients. There was insufficient evidence to suggest that the interventions affected treatment choice or anxiety [28]. There was substantial variation within outcomes between surgeons, emphasizing the usefulness of optimal and standardized implementation strategies.

Given evidence of the effectiveness of conversation aids, including the two from our parent trial, it is imperative to understand the factors and facilitators for routine implementation and sustainability of conversation aids in diverse clinical contexts for early-stage breast cancer, and particularly for patients of lower SES [29, 30]. While implementation and sustainability of conversation aids in routine clinical care has been evaluated in some contexts, the patient perspective has been notably absent in evaluation [29, 31–34]. In this study, we aimed to explore strategies that promote the conversation aids’ sustained use and dissemination using a theoretical implementation model. We also aimed to distinguish any differences in experiences and opinions between the two conversation aids and across varied socioeconomic strata.

## Methods

The protocol for the parent study, What Matters Most, is published elsewhere [35]. We conducted semi-structured interviews with What Matters Most patient participants, trial surgeons, and other relevant stakeholders to explore how two conversation aids can be implemented and sustained in diverse contexts in the future. All methods and results are reported using the COnsolidated criteria for REporting Qualitative (COREQ) research checklist (Additional file 1) [36].

### Interventions

The Option Grid conversation aid for early-stage breast cancer presents evidence-based information on breast-conserving surgery with radiation and mastectomy in a comparative table (Additional file 2) [37]. The Picture Option Grid conversation aid includes the same information as the Option Grid, but uses pictures and fewer words (Additional file 3). The Picture Option Grid was iteratively designed and developed using a Community Based Participatory Research (CBPR) approach [30, 38]. Pictures have been shown to improve comprehension of health information when closely linked with text or spoken words, and this relationship may be enhanced for individuals with lower health literacy [39–43]. The Picture Option Grid was designed for use with all patients diagnosed with early-stage breast cancer during the surgical consultation, but particularly for patients with lower health literacy and lower SES. Both interventions are paper-based with a sixth-grade readability level.

### Setting and participants

We recruited participants for interviews across seven clinics at four NCI-designated cancer centers in the United States with diverse patient populations. Three sites had urban and ethnically diverse populations, and one site had a rural, mostly white population. Two urban sites were specifically selected for recruiting patients of lower SES. Additional file 4 shows a table that summarizes usual care characteristics for each site.

We interviewed:

- All participating surgeons after trial involvement (*n* = 16). Prior to starting the trial, all surgeons underwent training that included information about the trial protocol, shared decision-making, and communication skills. Training for surgeons in intervention arms included videos and role plays on how to use their assigned conversation aid and when (during the surgical consultation). Surgeons in intervention arms were also provided feedback on their use of the conversation aid throughout the trial period, during scheduled supervision visits. As decided by the parent trial team (including patient partners), we included usual care surgeons to understand their experience in the trial and gauge their interest in using the conversation aids once the trial was over.
- A purposive sample of trial patient participants in intervention arms who agreed to be contacted for a 3-month post-operative interview. Family of invited patient participants were allowed to join the patient’s interview if they were involved when the intervention was used. Please see the parent trial protocol for participant inclusion criteria [35].
- Clinical and non-clinical stakeholders, including nurse practitioners, nurses, physician assistants, social workers, administrators, and electronic health record (EHR) specialists. Most interviewed clinical stakeholders and one non-clinical stakeholder were part of integrating the trial activities in the clinics where they worked. Some non-clinical stakeholders, in administration, provided initial support for the conversation aids’ use.

We planned to collect up to 60 patient interviews and 40 surgeon and stakeholder interviews [35]. We discussed data saturation as a core group (M-AD, RWY, CHS, DS, GE) as interviews were occurring, and with the broader parent trial team and stakeholders to reach consensus on when to stop conducting interviews. Retrospectively, we conducted a saturation test using Guest and colleagues’ approach to assess and report thematic saturation in qualitative research (*p* < 0.05 )[44].

### Theoretical framework and interview guides

We developed semi-structured interview guides in partnership with the parent trial’s surgeon, patient, and professional stakeholders as part of a CBPR approach [30]. There were separate interview guides for patients, intervention surgeons, usual care surgeons, clinical stakeholders, and non-clinical stakeholders (Additional file 5). We targeted a 30-min interview duration. We piloted all interview guides with team members, including patient partners (breast cancer survivors), and made necessary revisions, like making sure the interview flowed smoothly and reducing repetition, before starting data collection.

We developed the guides using the constructs and components of Normalization Process Theory (NPT). NPT is a framework that was developed to understand how complex interventions become implemented in routine healthcare settings using four theoretical constructs to contextualize implementation mechanisms [45, 46]. NPT has reliably been used to evaluate implementation and sustainability of similar conversation aids like Option Grid in the past. However, NPT has not been used to analyze the patient perspective on implementation and sustainability [31, 33, 34]. To our knowledge, there is no existing framework to analyze implementation from the patient’s perspective, so we adapted NPT to include the patient perspective (see Analysis). NPT’s four theoretical constructs include (1) Coherence: processes of individual and communal sense-making of a complex intervention regarding its use and value; (2) Cognitive participation: processes that promote or hinder users’ buy-in and commitment to the intervention; (3) Collective action: processes that determine whether the intervention is being used by all as intended; and (4) Reflexive monitoring: processes of communal and individual appraisal of the effect of the intervention [47].

We also developed the interview guides with a few hypotheses in mind, although not typically required in qualitative research [31, 48–51]. We used these hypotheses as probes used during the semi-structured interviews to explore their role in sustained use of the conversation aids. Our first hypothesis was that pre-visit planning, minimal clinician training, flexibility of use, and integration into the workflow and EHR would facilitate future sustained use. Our second hypothesis was that successful use by patients and families will be determined by the perceived acceptability of the intervention and integration into workflows. For full hypotheses, refer to the parent trial protocol [35].

### Procedure

#### Patient interviews

Research staff at each site conducted the interviews with patients. All interviewers were female and ranged in education from a bachelor’s degree to a master’s degree. Two study members (RWY and M-AD) trained research staff at each site in semi-structured interview techniques, such as probing and redirection, before conducting interviews [52].

Research staff approached previously-consented parent trial patient participants via phone call or in-person at a patient’s follow-up appointment in the clinic three months after the patient’s surgery. Research staff made no more than five attempts to contact participants to schedule an interview. After a research team member conducted their first interview, two members of the research team (RWY and M-AD) reviewed the transcript and provided feedback to the interviewer. Research staff conducted interviews over the phone or in-person depending on the interview participant’s preference and availability. Apart from recruiting the participants into the parent trial, there was no established relationship between the interviewers and the interview participants. We ensured interview participants had a copy of their assigned conversation aid before the interviews were conducted. All patient participants were compensated for their time with a $30 gift card. No repeat interviews were carried out.

#### Surgeon and stakeholder interviews

Surgeons consented to be contacted for the post-trial interview prior to recruiting patients for the parent trial. Stakeholders consented to the interview after being contacted post-trial. We chose a researcher external to What Matters Most (RF), a female PhD candidate with a master’s degree, to conduct the surgeon and stakeholder interviews to minimize positive response bias. The researcher conducted the interviews over the phone. All surgeon and stakeholder interview participants consented to their interview being audio-recorded. Surgeon and stakeholder interviewees were aware that their interviewer was not affiliated with the parent trial.

#### Observations and field notes

Research staff at each site were trained in ethnographically informed methods prior to recruiting participants for the broader study [53]. Research staff collected observational information from each site using an Observation Grid (designed to assist in observation using methods derived from ethnography), which includes sections to fill in information about physical observations in the clinic (e.g., waiting areas) as well as observations about the clinicians and patients (Additional file 6). The Observation Grid included sections on identifying materials in the clinics and chatter from clinic staff and patients about the study or the conversation aids. Additionally, research staff were encouraged to ask clinic staff questions about the study and conversation aids. Lastly, research staff were encouraged to write down their thoughts about what they observed and questions or concerns in a weekly email to the study coordinator (RWY) throughout the parent trial recruitment.

### Data management and analysis

#### Data management

Interview audio-recordings were transcribed verbatim by a HIPAA-compliant transcription service, Civicom Inc. For interviews that were conducted without a recording, the interviewer took in-depth notes on the interview guide for coding and analysis. The transcripts were not returned to participants for comments or corrections. While we did not check with the interview participants themselves, we confirmed our findings with the parent trial’s stakeholders, patient partners, and Community Advisory Board.

#### Analysis

We used a hybrid approach to our analysis, including framework and inductive analyses. Our main analysis was guided by NPT, however, we conducted an inductive analysis to allow for new codes to emerge. We also conducted analyses based on conversation aid used (Picture Option Grid versus Option Grid) and participant SES. For this, we stratified patients according to higher or lower SES by using their reported income level and number of dependents to calculate if they were above or below the 138% federal poverty level in the year of recruitment [54]. If income was not available, we used health insurance as a proxy where no insurance or government insurance without supplemental indicated lower SES, and private insurance or government insurance with supplemental indicated higher SES. We conducted *χ*^2^ tests and two-sample *t*-tests to determine if there were significant differences between those who declined versus not when approached.

We analyzed all interview transcripts, field notes, and observations using NPT through the lens of the NOrmalization MeAsure Development (NoMAD) instrument. The NoMAD instrument is a 20-item list organized by NPT constructs (Additional file 7) [55, 56]. NoMAD uses NPT constructs and components to assess participants’ opinions about how an intervention impacts their work and whether the intervention can be integrated and routinely used in their practice. The NoMAD items that correspond to NPT are composed of “I” statements like, “I can see the potential value of Option Grid/Picture Option Grid for my work.” Given NoMAD was not originally designed to evaluate implementation from the patient’s perspective, we adapted it for patient interviews. We adapted NoMAD to include the patient perspective by looking at the definition NoMAD provides for each NPT component and construct (for health professionals) and adapting item wording to focus on the patient experience. Adaptation occurred through collaboration between the two coders and arbitration by the senior author. See Table 1 for NoMAD’s definitions of each NPT construct and component for this study, including our addition of the patient perspective.

**Table 1:** Framework conceptualization from each stakeholder’s perspective

Construct ***component***	Surgeon and stakeholder definition	Patient definition
**Coherence — What is the work?**		
*Differentiation*	I can see how the Option Grid/Picture Option Grid differs from usual ways of working.	The Option Grid/Picture Option Grid seemed different/didnʼt seem different from other tools/things I’ve received since my diagnosis.
*Communal specification*	Staff in this organization have a shared understanding of the purpose of this Option Grid/Picture Option Grid.	*No codes came from this component.*
*Individual specification*	I understand how the Option Grid/Picture Option Grid affects the nature of my own work.	I understand how the Option Grid/Picture Option Grid affected the nature of my appointment with my surgeon.
*Internalization*	I can see the potential value of the Option Grid/Picture Option Grid for my work.	I'd recommend that other patients use this tool.
**Cognitive participation — Who does the work?**		
*Initiation*	There are key people who drive the Option Grid/Picture Option Grid forward and get others involved.	*No codes came from this component.*
*Legitimation*	How likely I am to recommend the tool to another health professional.	How likely I am to recommend the tool to a friend or family member.
*Enrollment*	I'm open to working with colleagues in new ways to use the Option Grid/Picture Option Grid.	How I hypothetically would like to receive the tool.
*Activation*	I will continue to support the Option Grid/Picture Option Grid.	I will continue to support the Option Grid/Picture Option Grid.
**Collective action — How does the work get done?**		
*Interactional workability*	I can easily integrate the Option Grid/Picture Option Grid into my existing work.	The Option Grid/Picture Option Grid was/was not well integrated into my visit with the surgeon. It seemed like the Option Grid/Picture Option Grid was a usual part of the appointment with my surgeon.
*Relational integration*	The Option Grid/Picture Option Grid disrupts working relationships. I have the confidence in other people's ability to use the Option Grid/Picture Option Grid.	*No codes came from this component.*
*Skill set workability*	Work is assigned to those with skills appropriate to the Option Grid/Picture Option Grid. Sufficient training is provided to enable staff to use the Option Grid/Picture Option Grid.	Work is assigned to those with skills appropriate to the Option Grid/Picture Option Grid.
*Contextual integration*	Sufficient resources are available to support the Option Grid/Picture Option Grid. Management adequately supports the Option Grid/Picture Option Grid.	*No codes came from this component.*
**Reflexive monitoring — How is the work understood?**		
*Systemization*	I am aware of reports about the effects of the Option Grid/Picture Option Grid on outcomes and workflows.	I understand how the Option Grid/Picture Option Grid affected me and my decision I had to make.
*Communal appraisal*	The staff agree that the Option Grid/Picture Option Grid is worthwhile.	*No codes came from this component.*
*Individual appraisal*	I value the effects the Option Grid/Picture Option Grid has had on my work.	I understand the effect the Option Grid/Picture Option Grid had on my surgeon's work.
*Reconfiguration*	Feedback about how the Option Grid/Picture Option Grid can be used to improve it in the future. I can modify how I work with the Option Grid/Picture Option Grid.	Feedback about how the Option Grid/Picture Option Grid can be used to improve it in the future.

Two researchers (DS and RWY) purposively selected six transcripts that were evenly divided between the type of interview (patient, surgeon, stakeholder) and coded them using both the NPT framework and descriptive codes to develop the codebook. The coders shared the codebook with two other team members (M-AD and GE) before continuing coding. One researcher coded a 20% purposive sample of the interviews (RWY) selected across types of interview (patient, surgeon, stakeholder) and study site. Another researcher (DS) coded all interview transcriptions, observations, and notes. Where there was disagreement between coders, a third individual helped the primary coders reach consensus (M-AD). All coding was conducted using ATLAS.ti. A research collaborator outside of What Matters Most with experience in NPT (PS) convened with the two coders to help determine the major and minor themes that emerged from the data once coding was completed.

## Results

### Participant characteristics

#### Patients

Forty-three of 76 patients approached (56.6%) agreed to be interviewed. Those who declined were more likely to be Hispanic, Spanish-speaking, have less education, and be of lower SES. Eighteen of the patients interviewed received the Option Grid in their surgical encounters, and 25 patients received the Picture Option Grid. Over half of patients were White and non-Hispanic and spoke primarily English. About a quarter of the patients were Black, non-Hispanic, and all others were either Hispanic or Asian. Fifty-one percent of patients had a 2-year college degree or higher. Approximately three-quarters of the patients were considered higher SES. Please see Table 2 for detailed patient participant characteristics, stratified by consent status. Imbalances in intervention received and socioeconomic status categories are consistent with the larger trial data.

**Table 2:** Patient participant characteristics, stratified by interview status

Characteristic	Interviewed (***n*** = 43)	Declined interview (***n*** = 33)	***p***-value*
**Age, mean (SD)**	56.6 (12.0)	62.4 (13.7)	0.05
**Race/ethnicity,** ***n*** **(%)**			0.04
Black, non-Hispanic	10 (23)	8 (24)	
Hispanic	2 (5)	9 (27)	
Asian	3 (7)	1 (3)	
White, non-Hispanic	28 (65)	15 (45)	
**Primary language,** ***n*** **(%)**			0.01
English	41 (95)	24 (73)	
Spanish	1 (2)	8 (24)	
Mandarin	1 (2)	1 (3)	
**Education,** ***n*** **(%)**			0.03
Never attended high school	1 (2)	3 (9)	
Some high school	0 (0)	6 (18)	
High school diploma (or equivalent)	11 (26)	6 (18)	
Some college	9 (21)	5 (15)	
2-year degree	6 (14)	6 (18)	
4-year degree or higher	16 (37)	7 (21)	
**SES,** ***n*** (%)**			0.01
Higher SES	32 (74)	15 (45)	
Lower SES	11 (26)	18 (55)	

*Chi-square tests used for categorical and dichotomous variables, t-tests used for continuous variables. Statistical tests had a null hypothesis of no difference in the distribution of the variables between interviewees and non-interviewees.

**Patients were considered lower SES if they were below 138% of the Federal Poverty Level based on income and household size for the calendar year they were enrolled in the trial.

#### Surgeons and stakeholders

All trial surgeons (n=16) participated in the interviews (five used Option Grid, six used Picture Option Grid, and five used usual care). The surgeons were majority female (81%), had an average of 23 years since graduating medical school, and an average of 10 years working at their current site. All surgeons had interest in shared decision-making prior to the study (see Table 3).

**Table 3:** Select characteristics of participating surgeons (*n* = 16)

Characteristic	
**Arm,** ***n*** **(%)**	
Option Grid	5 (31%)
Picture Option Grid	6 (38%)
Usual Care	5 (31%)
**Female sex,** ***n*** **(%)**	13 (81%)
**Years since graduating medical school, m (range)**	23 years (10-44)
**Years at current site, m (range)**	10 (< 1 to 30)
**Interest in SDM before trial**	16 (100%)

Thirty other stakeholders were contacted for an interview, of which 14 were interviewed. These 14 stakeholders included three nurse practitioners, three nurses, one physician assistant, one social worker, and six other non-clinical stakeholders that serve in administration or EHR-related roles.

### Setting and interview characteristics

Forty-nine interviews were conducted over the phone, 19 in clinic, and 5 in other locations (e.g., office). Four interviewees declined to be recorded, so the interviewer took detailed notes. One patient recording was lost due to technical difficulties. The average length of interviews for patients, surgeons, and stakeholders was 24, 27, and 20 min respectively. Please see Additional file 8 for the average length of interviews stratified by site and interviewee type. No individuals other than the interviewer and participant were present for the interviews.

### Major themes according to NPT construct

Table 4 summarizes the major themes for patients and surgeons organized by the four NPT constructs. All themes were developed in analysis from codes from interview transcripts, observations, and field notes.

**Table 4:** Summary of major themes according to the NPT constructs

NPT construct	Major themes
Coherence	Patients thought the Option Grids were easier to understand and more concise than other breast cancer materials. Using the Option Grids felt like usual care for surgeons.
Cognitive participation	Patients and surgeons recommended using Option Grids and were open to receiving and using them in various ways.
Collective action	Surgeons (after a few uses) and patients perceived the Option Grids were a part of a normal work routine.
Reflexive monitoring	Surgeons did not feel the Option Grids increased their consultation time.

The following sections describe the major themes in more detail.

#### Major themes related to coherence — “What is the work?”

Regardless of SES and intervention received, when asked, most patients felt the conversation aids were easier to understand and more concise compared with other breast cancer materials they had received as part of their cancer care (Differentiation).

“It was more concise. Other materials I received, you had to read through a whole bunch of things before you could get to the end and decide." — Patient, Option Grid, Lower SES

For most intervention surgeons, the use of the conversation aids felt similar to their usual care, in that the conversation aids included information they typically discuss with their patients (Differentiation). However, for both interventions, about half of surgeons thought the conversation aids disrupted the flow of their usual conversation. For example, some surgeons did not want to go in order that the material was presented on the conversation aid. For both interventions, half of the intervention surgeons and three other stakeholders felt that the conversation aids would be particularly helpful for patients with low health literacy or other associated factors like lower health literacy and limited formal education (Internalization).

When you're sitting there and actually showing them the picture and they can visualize what you're telling them, it does help, especially in our patient population where they don't necessarily understand everything we are saying to them. — Clinical stakeholder

#### Major themes related to cognitive participation — “Who does the work?”

For both interventions, approximately one-third of patients thought email or the patient portal would hypothetically be a helpful way to receive the conversation aid. Patients were open to various methods of receiving the conversation aids (email, mail, patient portal), but almost all highlighted the importance of paper-based versions (Enrollment). Regardless of SES or intervention received, almost all patients recommended that others like them use the conversation aids. They felt the conversation aids should be used in breast cancer care in the future (Legitimation).

I would give it to me on paper, but I would also send it to my email - it doesn’t have to be one way. It could be two ways, it could be three ways. — Patient, Picture Option Grid, Higher SES

Half of the surgeons thought it would be helpful if patients received the conversation aids prior to their surgical consultation. For both interventions and including usual care, about half of surgeons felt integrating the conversation aids in the electronic health record and/or the patient portal would be helpful (Enrollment). Most surgeons recommended that others like them should use the conversation aids (Legitimation).

I think having something beforehand, even if it’s just a heads-up, would be helpful. — Surgeon, Option Grid

#### Major themes related to collective action — “How does the work get done?”

Regardless of SES or intervention received, over half of patients thought their surgeons used the conversation aid with ease and it appeared part of their normal routine (Interactional workability).

*...*because it flowed into our appointment so seamlessly. It definitely seemed like part of how [the surgeon] would present the information. — Patient, Picture Option Grid, Higher SES

Most intervention surgeons had to use the conversation aid a few times before using it with ease. After a few uses, they felt the conversation aid became part of their normal routine (Interactional workability). For both interventions, all surgeons thought the training on shared decision-making and how to use their assigned intervention was sufficient and did not recommend any changes (Skillset workability).

Well, it became pretty much second-hand for me actually. I got really used to using it. It just became part of my routine. — Surgeon, Picture Option Grid

#### Major themes related to reflexive monitoring (“How is the work understood?”)

For both interventions, over half of patients felt the conversation aid affected their treatment decision (Systemization).

I had a lumpectomy instead of a mastectomy because the information contained in the grid helped me understand that I didn’t need one [mastectomy]. — Patient, Picture Option Grid, Higher SES

Surgeons understood the conversation aids were designed to help structure their conversation with eligible patients and help them make a treatment decision. For both interventions, all surgeons felt that the time it took to use the conversation aid did not change the typical time they spend with patients in the surgical consultation (Individual appraisal).

*...*as I got faster, I got more used to it. It did help the rhythm. At first, it took me a little bit longer than I think it would’ve been, but not much. Then, at the end, I think it ultimately helped structure things. It might have made things as efficient or more efficient. — Surgeon, Option Grid

See Additional file 9 for a detailed analysis including major and minor themes from all types of stakeholders (patients, surgeons, other stakeholders) according to NPT constructs and components.

### Comparing major themes by participant SES

Patients of higher SES, for both interventions, were more likely to find the conversation aid easier to understand than materials they had received before. They found the conversation aid concise and understood it was designed to help them compare their treatment options (Coherence). For patients of lower SES in the Picture Option Grid group who found the conversation aid easy to understand, all found the pictures played a significant role in their understanding (Coherence). Patients of lower SES who received Picture Option Grid and patients of higher SES who received Option Grid had a preference for paper-based conversation aids. Patients of higher SES reported more willingness to hypothetically receive the conversation aid ahead of their appointment (for example, through the patient portal) compared with patients of lower SES, who were more likely to mention that receiving it directly from their surgeon would be best (Cognitive participation). Patients of higher SES, for both interventions, mentioned more often that the conversation aid affected the discussion with their surgeon compared with patients of lower SES were more likely to mention it influenced their treatment decision (Reflexive monitoring).

I interacted with the piece of paper a hundred times more than I would with the downloaded file if somebody had just sent that to me to look at before my appointment. — Patient, Option Grid, Higher SES

I’d say it really influenced it [my decision]. — Patient, Option Grid, Lower SES

I would recommend it to everybody. — Patient, Option Grid, Lower SES

Additional file 10 features a detailed analysis comparing both major and minor themes by participant SES. Additional files 11 and 12 include a detailed analysis with both major and minor themes comparing SES for each intervention.

### Comparing major themes by intervention

Patients who received Picture Option Grid reported more willingness to hypothetically receive the conversation aid in advance of their appointment. Patients who received Option Grid were more likely to mention that receiving the conversation aid from their surgeon, and paper-based, would be best (Cognitive participation). Patients who received Option Grid found that using the conversation aid did not feel awkward (Collective action). Patients who received Option Grid were slightly more likely to feel the conversation aid influenced their discussion with their surgeon (Reflexive monitoring).

Completely influenced [my decision] — Patient, Option Grid, Higher SES

It needs to be something they can see online in MyChart. It should also be in a doctor’s office so that a woman can, you know, “Let me read this.” — Patient, Picture Option Grid, Lower SES

All surgeons who used Option Grid felt using the conversation aid did not feel different from their usual practice compared with only half of surgeons who used Picture Option Grid (Coherence). Surgeons who used Picture Option Grid were more likely to mention the conversation aid should be given at the surgical consultation, as was done in the RCT. They were also more comfortable with their nurse or other qualified clinic personnel giving the conversation aid (Cognitive participation). Surgeons who used Picture Option Grid found they were able to integrate the conversation aid into their normal practice, especially after a few uses (Collective action). Some Picture Option Grid surgeons found the pictures not helpful, but most had no concerns with patients using the conversation aid.

Well, it became pretty much second-hand for me actually. I got really used to using it so I don’t know. It just became part of my routine. — Surgeon, Picture Option Grid

Additional file 13 includes a detailed analysis comparing both major and minor themes by intervention used or received.

## Discussion

### Main findings

The future implementation of the early-stage breast cancer Option Grid conversation aids across diverse practices shows promise for sustainability. Key facilitators include adapting the conversation aids to existing clinical workflows, using them in an adaptable manner that fits best within a surgeon’s consultation, and recognizing patient characteristics and communication preferences. No major barriers were reported by patients, surgeons, or stakeholders in sustainably implementing these conversation aids in diverse practices.

Over half of patients thought their surgeons used the conversation aids as part of their usual care. Almost all recommended others like them use the conversation aids. Patients of higher SES were more likely to note the conversation aids influenced their treatment discussion, while patients of lower SES noted more influence on their decision-making. Patients were open to receiving the conversation aids in various ways (EHR, patient portal, email, prior to appointment). For all intervention surgeons, using the conversation aids did not change the typical time they spent with their patients. Most intervention surgeons felt using the conversation aids enhanced their usual care (in that they were already engaging patients in treatment option discussion), became part of their normal routine, and took a few times before using the conversation aid with ease. Most surgeons recommended other surgeons like them use these conversation aids in the future. Overall, there were no noted major downsides to using the conversation aids, thus facilitating future adoption and potential sustainability of these conversation aids in diverse clinical settings.

### Strengths and limitations

A strength of our study is the large number of interviews conducted with a diverse group of stakeholders. We had diversity in type of interviewee (patient, surgeon, stakeholder) and in demographics (race, SES, health literacy). A researcher not affiliated with What Matters Most conducted interviews with the surgeons and stakeholders to minimize positive response bias. Using NPT with multiple perspectives provided a holistic picture of the implementation and sustainability factors of the conversation aids. As a novel approach, we analyzed the patient perspective using NPT as it is important to understand patients’ opinions regarding the implementation of conversation aids with diverse patient groups.

One limitation is the discrepancy in patient interview lengths across sites potentially resulting in more site representation than others. However, the same training materials, protocols, and transcript review mechanisms were used across all sites to promote consistency and quality control. There were significant differences between patients who agreed to the interview compared with those who declined, which might limit the generalizability of our findings. Decliners were more likely to be Hispanic and Spanish-speaking, although they were offered to complete the interviews in Spanish with a translator present. Decliners were also more likely to have less education and be of lower SES. Further, our results should be interpreted with caution as we conducted an implementation and sustainability assessment using an implementation framework in a comparative effectiveness trial [57]. There may also be selection bias from our surgeons as all surgeons had interest in shared decision-making prior to recruiting for the broader trial. Surgeons and clinical staff were not asked to routinely integrate these conversation aids in their clinical practices. We cannot expect that the opinions and thoughts generated from these interviews were always directly related to implementation and sustainability factors.

### Results in context

Our findings align with existing evidence, including in early-stage breast cancer, that it takes flexibility of use and a few tries for a clinician to become comfortable using a conversation aid [15, 49]. While previous barriers to implementation have noted surgeons’ concern regarding conversation aids increasing encounter duration, our findings align with a previous study suggesting surgeons did not report an impact of the conversation aids on the length of their regular consultation [58].

Surgeons who were able to integrate the conversation aid in their clinical practice mentioned the conversation aid was similar to their usual consultation content, also seen in a similar study on contraceptive counseling [17]. Some patients, surgeons, and stakeholders were interested in integrating the conversation aids in the EHR. However, the sample was divided with emphasis on the importance of paper-based conversation aids. Our findings align with Politi et al.’s study that examined clinicians’ use of tools where there is no “one-size-fits-all” approach to implementing conversation aids, with variation in patient preferences in format and clinicians’ opinions about conversation aids [59].

Our results coincide with another study on a different surgical conversation aid that most patients mentioned the best place to use the conversation aids was during the surgical consultation [60]. However, as also seen in Bunzli’s study, patients mentioned that receiving the conversation aid beforehand would be helpful in some circumstances provided the patient already knew their diagnosis [60]. Patients also noted that the conversation aids covered relevant content and the information provided was helpful and served as a helpful starting point. Our findings that patients felt the conversation aids covered relevant content and served as a starting point coincides with Bozic’s study findings on orthopedic surgery decision and communication aids [61].

According to patients and surgeons who liked using the conversation aids, they mentioned that Picture Option Grid appeared particularly powerful with the inclusion of images. Research shows that pictures in health communication may help better facilitate knowledge for disadvantaged patients than words alone [39–43]. A systematic review showed pictures in health information moderately improved knowledge and recall, but largely increased knowledge for lower health literacy populations [62]. It is imperative to address patients with lower health literacy or other health inequities by developing and using conversation aids that address their needs [63]. Individual patient characteristics and the health system that they are familiar interacting with is an important determination for which conversation aid will be most helpful.

### Implications

Barriers to implementing similar conversation aids continue to exist despite a general understanding that these conversation aids are helpful for patients [64–66]. We found in our study that individual patient characteristics, like health literacy level and communication preferences, is important in determining which conversation aid will be most appropriate to use. However, clinicians’ discretion may not suffice, in which case, patients should be offered a choice between the two conversation aids or given both. Healthcare professionals’ willingness to use and confidence in the conversation aid is a facilitator to implementation. Those willing to integrate a conversation aid must do so by adapting it to their existing workflow and clinical environment to ensure sustained implementation. With a small learning curve and adjustment to workflow, surgeons we interviewed said that using the conversation aid did not seem to extend the consultation time, and they reported no major downsides to using them. This study exemplifies that commonly noted barriers to implementation and sustainability can be overcome with enough willingness to improve healthcare delivery from the patient, clinical, and organizational standpoints.

## Conclusion

This study is novel in our effort to understand the experiences and opinions of implementation and sustainability factors of two conversation aids for early-stage breast cancer with multiple stakeholder perspectives, including the patient perspective. We found that in order to successfully implement and ensure sustainability, the conversation aids must be skillfully adapted to clinical workflows and integrated flexibly in health professionals’ usual care. Individual patient characteristics, like health literacy and existing knowledge about breast cancer, and communication preferences are important factors in determining which conversation aid to use. However, we also recommend that patients are given a choice between the two or given both. Patients and surgeons agreed that the conversation aids should be used in breast cancer care in the future and were open to various methods of giving and receiving the conversation aid, allowing for adaptability of future implementation efforts. In this study, the future implementation of the early-stage breast cancer Option Grid conversation aids across diverse clinical practices shows promise for sustainability.

## Supplementary Information

**Additional file 1.**

**Additional file 2.**

**Additional file 3.**

**Additional file 4.**

**Additional file 5.**

**Additional file 6.**

**Additional file 7.**

**Additional file 8.**

**Additional file 9.**

**Additional file 10.**

**Additional file 11.**

**Additional file 12.**

**Additional file 13.**

## Data Availability

The dataset analyzed during the current study is not publicly available due to the protection of the human subjects involved in the qualitative interviews. A deidentified copy of the dataset is available from the corresponding author on reasonable request.
