# Supplementary material for: Implementation and sustainability factors of two early-stage breast cancer conversation aids in diverse practices
Source: Implement Sci. 2021 May 10;16:51. doi: 10.1186/s13012-021-01115-1 (PMC8108365; doi:10.1186/s13012-021-01115-1)
Supplement: Supplementary file 2 — Additional file 2. [file 13012_2021_1115_MOESM2_ESM.pdf]

Breast cancer: surgical options

Use this **Option Grid™** decision aid to help you and your healthcare professional talk about how to best treat your breast cancer. This decision aid is for women with early stage breast cancer (stages I to IIIA).

Frequently asked questions	Lumpectomy with radiation	Mastectomy
**What is removed?**	The cancer lump is removed, with some surrounding tissue.	The whole breast is removed.
**Which surgery is best for long-term survival?**	Long-term survival rates are the same for both surgeries.	Long-term survival rates are the same for both surgeries.
**What are the chances of cancer coming back in the breast?**	Breast cancer will come back in the breast in about 5 to 10 in 100 women (5-10%) in the 10 years after a lumpectomy.	Breast cancer will come back in the area of the scar in about 5 to 10 in 100 women (5-10%) in the 10 years after a mastectomy.
**Will I need more than one surgery?**	Possibly, 20 in 100 women (20%) may need another surgery to remove breast tissue or lymph node that have cancer.	Possibly, if your lymph nodes have cancer.<br>Yes, if you choose breast reconstruction.
**How long will it take to recover?**	Most women are home within 24 hours of surgery.	Most women are home within 24 hours of surgery. It may take longer with reconstruction.
**Will I need radiation after surgery?**	Yes, for up to seven weeks after surgery.	Radiation is not usually given after mastectomy.
**Will my lymph nodes be removed?**	If cancer has spread to the lymph nodes under your arm, your doctor will discuss with you whether you need more treatment such as surgery or radiotherapy.	If cancer has spread to the lymph nodes under your arm, your doctor will discuss with you whether you need more treatment such as surgery or radiotherapy.
**Will I need chemotherapy?**	You may be offered chemotherapy, but this does not depend on the surgery you choose.	You may be offered chemotherapy, but this does not depend on the surgery you choose.
**Will I lose my hair?**	Hair loss is common after chemotherapy.	Hair loss is common after chemotherapy.

Editors: Marie-Anne Durand (Lead Editor), Glyn Elwyn, Lisa Caldon, Kari Rosenkranz, Dale Collins Vidal, Stephanie Sivell, Malcolm Reed
Evidence document: http://optiongrid.org/admin/resources/grid/evidences/9.pdf?x=WBZ1nd4YE
This Option Grid does not constitute medical advice, diagnosis, or treatment. See Terms of Use and Privacy Policy at www.optiongrid.org.
Publication date: 23 August 2017 Expiry date: 23 August 2019

Cáncer de mama: opciones quirúrgicas

Use esta herramienta de toma de decisiones **Option Grid™** para ayudarle a usted y a su profesional de la salud a conversar sobre cuál es el mejor tratamiento para su cáncer de mama. Esta herramienta de toma de decisiones es para mujeres con cáncer de mama en etapa temprana (Etapa I a IIIA).

Preguntas frecuentas	Tumorectomía con radiación	Mastectomía
**¿Qué se extirpa?**	Se extirpa el tumor cancerígeno, junto con algunos tejidos normales que lo rodean.	Se extirpa toda la mama.
**¿Qué cirugía es mejor en términos de supervivencia a largo plazo?**	Las tasas de supervivencia a largo plazo son las mismas para ambas cirugías.	Las tasas de supervivencia a largo plazo son las mismas para ambas cirugías.
**¿Cuáles son las probabilidades de que vuelva a tener cáncer de mama?**	El cáncer de mama reaparece en aproximadamente 5 a 10 de cada 100 mujeres (5-10%) en los 10 años siguientes a la Tumorectomía.	El cáncer de mama reaparece en el área de la cicatriz en aproximadamente 5 a 10 de cada 100 mujeres (5-10%) en los 10 años siguientes a la mastectomía.
**¿Podría necesitar más de una cirugía?**	Es posible. 20 de cada 100 mujeres (20%) pueden necesitar otra cirugía para extirpar el tejido mamario o ganglios linfáticos con cáncer.	Es posible, si sus ganglios linfáticos tienen cáncer. También podría necesitar otra cirugía si decide realizarse una reconstrucción mamaria.
**¿Cuánto tardaré en recuperarme?**	La mayoría de las mujeres regresan a su casa dentro de 24 horas después de la cirugía.	La mayoría de las mujeres regresa a su casa dentro de 24 horas después de la cirugía. Si se realiza una reconstrucción mamaria podría tomar más tiempo.
**¿Necesitaré radioterapia después de la cirugía?**	Sí, por hasta siete semanas después de la cirugía.	No se suele hacer radioterapia después de una mastectomía.
**¿Se extirparán mis ganglios linfáticos?**	Si el cáncer se ha propagado a los ganglios linfáticos debajo de su brazo, su médico conversará con usted sobre la necesidad de algún otro tratamiento, como cirugía o radioterapia.	Si el cáncer se ha propagado a los ganglios linfáticos debajo de su brazo, su médico conversará con usted sobre la necesidad de algún otro tratamiento, como cirugía o radioterapia.
**¿Necesitaré quimioterapia?**	Se le puede ofrecer quimioterapia, pero esto no depende de la cirugía que elija.	Se le puede ofrecer quimioterapia, pero esto no depende de la cirugía que elija.
**¿Perderé mi cabello?**	La pérdida de cabello es común después de la quimioterapia.	La pérdida de cabello es común después de la quimioterapia.

Editores: Marie-Anne Durand (Editor principal), Glyn Elwyn, Lisa Caldon, Kari Rosenkranz, Dale Collins Vidal, Stephanie Sivell, Malcolm Reed
Documento de prueba: http://optiongrid.org/admin/resources/grid/evidences/9.pdf?x=WBZ1nd4YE
Fecha de publicación: 23 agusto 2017 Fecha de vencimiento: 23 agusto 2019 ISBN: 978-0-9550975-6-0 Licencia: CC BY-NC-ND 4.0 (Internacional)
Este Cuadro de opciones no constituye un consejo médico, diagnóstico o tratamiento. Consulte los Términos de uso y la Política de privacidad en www.optiongrid.org.

乳腺癌：外科选择

使用 **Option Grid™** 来帮助您和您的医护专业人员讨论最合适的治疗方案。此文件是给患有早期乳腺癌 (I 至 IIIA 期) 的女性使用的。

常见问题	乳房肿瘤切除和放射治疗	乳房切除术
**切除什么？**	切除癌症肿块以及周围的一些组织。	切除整个乳房。
**哪种手术是长期生存的最佳选择？**	这两种手术的长期存活率是一样的。	这两种手术的长期存活率是一样的。
**乳腺癌复发几率是多少？**	在乳房肿瘤切除术后的 10 年中，每 100 名女性中约有 5-10 名（5-10%）会复发乳腺癌。	在乳房切除术后的 10 年中，每 100 名女性中约有 5-10 名（5-10%）的乳腺癌会在疤痕部位复发。
**我将需要多次乳房手术吗？**	有可能，100 名女性中有 20（20%）名可能需要再次手术，切除更多患有癌症的乳腺组织或淋巴结。	有可能，如果您的淋巴结有癌症。<br>是的，如果您选择乳房再造。
**恢复需要多长时间？**	大多数女性在手术后 24 个小时内就可以回家。	大多数女性在手术后 24 个小时内就可以回家，如果选择乳房再造则需要更长的时间。
**我在手术後会需要放射治疗吗？**	是的，手术后最多持续七周。	乳房切除术后通常不进行放射治疗。
**我的淋巴结会被切除吗？**	如果癌症扩散到您腋下的淋巴结，您的医生会和您讨论是否应考虑进一步的治疗，如手术或放射治疗。	如果癌症扩散到您腋下的淋巴结，您的医生会和您讨论是否应考虑进一步的治疗，如手术或放射治疗。
**我会需要化学治疗吗？**	可能向您提供化学治疗，但这并不取决于您选择的手术方案。	可能向您提供化学治疗，但这并不取决于您选择的手术方案。
**我会脱发吗？**	化学治疗后脱发是常见的。	化学治疗后脱发是常见的。

编辑: Marie-Anne Durand (Lead Editor), Glyn Elwyn, Lisa Caldon, Kari Rosenkranz, Dale Collins Vidal, Stephanie Sivell, Malcolm Reed
证据文件: http://optiongrid.org/admin/resources/grid/evidences/9.pdf?x=WBZ1nd4YE
此 Option Grid™ 并不构成医疗建议、诊断或治疗。 请参阅 使用条款 和 隐私政策, 位于 www.optiongrid.org。
出版日期： 23 August 2017 **到期日期：** 23 August 2019
