# Supplementary material for: Implementation and sustainability factors of two early-stage breast cancer conversation aids in diverse practices
Source: Implement Sci. 2021 May 10;16:51. doi: 10.1186/s13012-021-01115-1 (PMC8108365; doi:10.1186/s13012-021-01115-1)
Supplement: Supplementary file 4 — Additional file 4. [file 13012_2021_1115_MOESM4_ESM.docx]

**Appendix 4. Usual care characteristics at each site**

| **Site #** | **Setting characteristics** |
| --- | --- |
| 1 | - Emmi video about breast cancer surgery options mailed to patients after their diagnosis - Breast Cancer Treatment Handbook mailed to patients after their diagnosis - PowerPoint slides showing the breast and surgical treatment options |
| 2 | - Posters and information sheets displayed on cork boards - Educational pamphlets in waiting room and hallways - Patient navigator gives patient an instruction sheet for surgery and booklet on breast cancer |
| 3 | - Video clip presenting the surgical options shown during consultations. Available in English, Spanish and Mandarin - Posters representing the breast displayed in clinics - Notebook given to patients with information about breast cancer, surgical options, and other resources |
| 4 | - Educational flyers in waiting room and clinic room - Binders given to patients with American Cancer Society content |
