# Supplementary material for: Implementation and sustainability factors of two early-stage breast cancer conversation aids in diverse practices
Source: Implement Sci. 2021 May 10;16:51. doi: 10.1186/s13012-021-01115-1 (PMC8108365; doi:10.1186/s13012-021-01115-1)
Supplement: Supplementary file 5 — Additional file 5. [file 13012_2021_1115_MOESM5_ESM.pdf]

AIM 3: Explore strategies that promote the encounter decision aids' sustained use and dissemination using a theoretical implementation model.

Hypothesis 1. Pre-visit planning, minimal clinician training, flexibility of use, and integration into the workflow and EMR will facilitate sustained use.

Hypothesis 2. Successful use by patients and their families will be determined by the perceived acceptability of the intervention and integration into workflows.

++

Notes on interview probing

Please always remember that if you are not getting clear answers to your questions, use the following probes (or your own) to get the content and answer to the question you have just asked:

- In what way?
- Could you tell me more?
- Why or why not?
- Help me understand what you mean by that?

++

Notes on redirection

Here are a few examples on how to redirect the interviewee if she begins to go off topic and away from the content of the interview:

- Acknowledge what the participant has said then redirect the conversation.
- Rephrase what the participant said to acknowledge her thoughts then can say "let's take a step back" and then repeat the question
- Can say thank you for sharing if it feels appropriate

++

Interview guide begins on the next page.

Introduction

Hi, my name is ____________. Thank you for being here today. We would like to talk to you about the decision tool you received in the Matters Most trial. We want to learn more about how you used this tool with your doctor, care team, and afterward at home. It's important for you to know that this conversation is part of the research we are doing. We are not questioning the care you received in any way.

If taking notes: I will be typing some notes while we talk, and I just wanted to let you know that.

Do you have any questions for me before we begin?

Review any questions they have.

We would like to record this conversation so that we can make sure we capture everything you say. If you agree to this, we will remove anything that could identify you by name when we make the recording into a written document. Are you comfortable with me audio recording our conversation?

Based on the answer, turn the recorder on. Check to be sure the recorder is on. Proceed with the interview.

Icebreaker

First, how are you doing?

Have the correct tool in front of you as you ask the questions below.

Patient Associate Involvement

Do you remember meeting [*patient associate name*]?

Probe: What did you think about meeting her?
Probe: Did [*patient associate name*] tell you she was a breast cancer survivor?

Probe: Did that affect your participation in the study? If so, how?

Part 1: POG/OG Integration

In these first set of questions, I will ask you about your use of the tool.

a. **First, how did [*clinician name*] introduce the tool to you?** *[NPT - Collective action/Interactional workability]*

Probe: Who introduced it first?
NOTE: use the term for clinician or the clinician's name that the patient uses moving forward. [Only use if there's some question about who introduced it.]

Probe: Where was it introduced to you?
NOTE: adjust the term 'visit' in the following questions if the tool was introduced at a different point.

Probe: Were you sitting on the exam table?

Probe: Was there a table in front of you?

Probe: Did you hold the tool in your hands?

Probe: At what point in the clinic visit was the tool introduced to you?

b. **Did this seem like the right or wrong time to get the tool?**

c. **In your own words, what was the purpose of [*clinician name*] showing you the tool?**

Probe: How was the tool meant to help you?

d. **Did [*clinician name*] explain why they were using the tool with you?**

Probe: If yes, what did they say about it?

e. **How was the tool used during your visit?**

Probe for clarification if necessary, e.g., and what happened next?

f. **On a scale from 0 to 10 how hard was the tool to understand compared to other materials you received with 0 being much easier and 10 being much harder?**

Probe: Why?

Probe: Does the tool feel different to you from other materials you have received for your breast cancer treatment? *[NPT - Sense-making/Differentiation]*

Probe: Does the tool provide different information compared to other materials you received when you learned you had breast cancer?

g. **On a scale of 0 to 10 how comfortable did [*clinician name*] seem using the tool with 0 being not at all comfortable and 10 being very comfortable?** *[NPT - Cognitive participation]*

Probe: Why?

When [*clinician name*] used the tool with you, did you feel like it was a part of what she normally does? *[NPT - Sense-making]*

h. **Did anything feel awkward to you when using the tool?**

i. **Was there someone else with you at your clinic visit with the doctor?**

Probe: If yes, who?

Probe: If yes, did they look at the tool? What were their reactions? *[NPT - Cognitive participation/Enrolment]*

j. **Did you write on the tool during the clinic visit?**

Probe: If yes, tell me about what you wrote on it.
Probe, if necessary: Why?

k. **Did you take the tool home?**

Probe: If yes, how did you use it once it was at home with you?

l. **Did you show the tool to anyone after you first used it with the doctor?** *[NPT - Collective action]*

Probe: If yes, with who?
Probe: How did you use it with them?
Probe: What were their reactions?

m. **How many times did you use the tool after the clinic visit?** *[NPT - Sense-making]*

If the participant says never or only once, ask: Why?

Probe: Where did you use it? (prompts if needed: at clinic, at home, with counselor, etc.) *[NPT - Collective action]*

Part 2: Acceptability of the Picture Option Grid/Option Grid

Thank you for answering those questions. We're going to switch gears now and discuss your feelings about the tool itself.

n. **Tell me about your overall impression of the tool?**

Probe: What did you think about it?
Probe: What stands out about the tool?

o. **What did you dislike about it?**

Probe: (if others used the tool) What did others dislike about the tool?

p. **What did you like about it?**

Probe: (if others used the tool) What did others like about it?

q. **On a scale of 0 to 10, how difficult was it for you to *use* the tool with 0 being least difficult and 10 being most difficult?**

Probe: Why?
Probe: What did you think about the fact it was on paper?
Probe: Was the font easy to read?
Probe: Was it unhelpful? Helpful?
Probe: Was it difficult or easy for you to use the tool with others (family, friends)?

r. **On a scale of 0 to 10, how difficult was it for you to *understand* the information in the tool with 0 being least difficult and 10 being most difficult?**

Probe: Why?
Probe: Were there words that were confusing?
Probe: (POG only) Were there images that were confusing?
Probe: Were there numbers that were confusing?
Probe: Were the images representing the numbers confusing?
Probe: Was it difficult or easy to understand for others (family, friends)?

Part 3: Surgery decision-making

Thank you. We are going to switch gears again. I'm going to now ask you a few questions about how you made your decision between lumpectomy and mastectomy.

s. **On a scale of 0 to 10, how much did the tool influence your discussion with [*clinician name*] or care team about the choice you had between lumpectomy and mastectomy where 0 means not at all and 10 means completely influenced the discussion?** *[NPT - Monitoring/Systematization]*

   Probe: How did it influence your discussion?
      Probe: Did it influence the way you understood your surgery options?
   Probe, if needed: Did you use to the tool when you discussed your surgery options ***during*** the consultation?
   Probe, if needed: Did you use to the tool when you discussed your surgery options ***after*** the consultation?

t. **Do you feel [*clinician name*] wanted you to choose one option over another?**

   Probe: Why did you feel that way?

u. **In the end, on a scale of 0 to 10 how much did the tool influence how you made your decision with 0 being no influence and 10 being completely influenced?**

   Probe: How did it influence your experience?

Part 4: POG/OG Implementation

Thank you. I'm now going to ask about future use of the tool, by other women with breast cancer. I'd like to know how you think it should be used. This is the last set of questions.

v. **On a scale of 0 to 10, how likely are you to recommend this tool to a friend or family member with 0 being definitely will not recommend and 10 being definitely will recommend?** *[NPT - Monitoring] [NPT - Sense-making/Internalization]*

Probe: Why?

w. **Do you think this tool should be used in breast cancer care in the future?** *[NPT - Action]*

Probe: Why or why not?

x. **Do you have any concerns about other women like you using this tool?**

Probe: If yes, what are your concerns? Please explain them.

y. **Where do you think is the best place to use the tool? For example, in the doctor's office, as happened in this trial? In the waiting room before you see the doctor? Or, at home?**

Probe, if not already mentioned: Who should women use this tool with?

Probe, if not already mentioned: When do you think is the best time to use the tool?

Probe: Before the clinic visit? In the waiting room? At home before the visit?
Probe: During the clinic visit only?
Probe: After the clinic visit?
Probe: Before, during and after?

z. **There are many different ways that women could receive this tool. In your opinion, how should women receive it?**

Let her respond then go through the following options if not mentioned:

Given by your doctor during the visit, as was done for you?
Given by a nurse before the visit?
Sent via email?
Accessible on the patient portal (*can give example for your hospital system, e.g., eD-H*)?
Included in the after visit summary?
Included in your electronic health record?

aa. What would be the best way to use the tool with family members or friends?

bb. What would you change about how the tool is used with other women in the future?

Wrap Up

Thank you for your great feedback today. Your comments are very valuable to our research. Is there anything else you'd like to share about what we have discussed before we finish up this conversation?

Allow her to give any additional feedback.

Again, thank you for your time today and for your continued involvement in the What Matters Most study. Here is the $15 gift card for your participation today. *Or, I will mail you your $30 gift card for your participation today.*

If in-person, hand the interviewee their gift card.

We'll be in touch when [look up T5 month] when your last survey for the study will happen.

AIM 3: Explore strategies that promote the encounter decision aids' sustained use and dissemination using a theoretical implementation model.

Hypothesis 1. Pre-visit planning, minimal clinician training, flexibility of use, and integration into the workflow and EMR will facilitate sustained use.

Hypothesis 2. Successful use by patients and their families will be determined by the perceived acceptability of the intervention and integration into workflows.

++

Notes on interview probing

Please always remember that if you are not getting clear answers to your questions, use the following probes (or your own) to get the content and answer to the question you have just asked:

- In what way?
- Could you tell me more?
- Why or why not?

++

Notes on redirection

Here are a few examples on how to redirect the interviewee if s/he begins to go off topic and away from the content of the interview:

- Acknowledge what the participant has said then redirect the conversation.
- Rephrase what the participant said to acknowledge her thoughts then can say "let's take a step back" and then repeat the question.
- Can say thank you for sharing if it feels appropriate.

++

Interview guide begins on the next page.

Introduction

Hi, I'm ____________. Thanks for taking the time to speak with me today about your involvement in the Matters Most trial and your use of the *[Option Grid/Picture Option Grid]*. Although I am a part of The Dartmouth Institute, I'm not directly involved in the trial. I hope you'll feel free to be as open in our discussion as possible.

The information sheet I emailed to you earlier is primarily intended to let you know that 1) today's discussion is for research purposes; 2) participation is voluntary and you can skip any questions that you'd like; and 3) the information you discuss will be kept confidential won't be attributed to you by name in any reports or presentations about this project.

Do you have any questions before we begin?

Review any questions they have.

We'd like to record this conversation so that we can capture everything you say. We'll remove anything that could identify you by name. Are you comfortable with me audio recording our conversation?

Based on the answer, turn the recorder on. Check to be sure the recorder is on. Proceed with the interview.

Take detailed notes if they say no to recording.

If the surgeon was in the intervention arm, have the correct tool in front of you as you ask the questions. Please only talk about the correct intervention and replace POG/OG by the correct term: Picture Option Grid or Option Grid (depending on randomization).

Part 1. POG/OG Use and Acceptability

a. **What do you think of the POG/OG?**

Probe: What stands out about it?
Probe: What did you like?
Probe: What did you dislike?
Probe: What did the patients think about it? What about others in the room?
Probe: What do others in your team think about it?

b. **I know you've been asked this before but could you walk me through how you used it.**

Probe: What was it like to use it for an extended period of time?
Probe: What did it feel like to be the one who introduced it to patients?
[NPT: Cognitive participation - Legitimation]
Probe: If relevant, what was it like for your team?
Probe: Did you always use it in the same way?
Probe: Were there differences from your usual practice?
[NPT: Coherence - Differentiation]
Probe: Did you use it differently with different types of patients?

Probe: For example, patients of different SES or race?

Probe: Did you write on it? (if not covered before)

c. **How difficult was it to use the POG/OG with 0 being least difficult and 10 being most difficult?**

Probe: Did it change over time?
Probe: What about the time it takes to use it?

Part 2. Your Involvement in the Matters Most Trial

a. **We talked about POG/OG specifically but overall, what was it like to take part in the Matters Most study?** *[NPT: Coherence - Individual specification]*

Probe: The goal of the trial was to involve patients in decisions using tools, any comments?
Probe: What was it like for your team?
[NPT: Coherence - Communal specification; Cognitive participation]
Probe: Was it challenging? If yes, how, and why?
Probe: On a scale of 0 to 10 with 0 meaning no effect, how much did the study affect the clinic workflow?
Probe: How, and why?
Probe: Did you benefit from participating in the study? If yes, how, and why?
[NPT: Coherence - Internalization; Reflexive monitoring]

b. **(if not covered in answers to question a) Did you have to make changes to comply with the study procedures?**
[NPT: Coherence - Differentiation & Collective action]

c. **(If not covered in answers to question a) Did your team have to make changes to comply with the study procedures?**
[NPT: Coherence - Differentiation]

d. **What were your interactions like with the research team?**

Probe: Was it easy or difficult to work with the research team?
Probe: Did you encounter [*name of patient associate*], the patient associate?
Probe: What was it like to work with [*name of patient associate*]?
Probe: In general, what would you change about the involvement of a patient associate?

e. **Could the study team have improved surgeon and team involvement in the study?**
[NPT: Coherence - Reconfiguration]

Probe: If yes, how?

f. **On a scale of 0 to 10 how likely are you to take part in a study like Matters Most again with 0 being least likely and 10 being most likely?**
[NPT: Reflexive monitoring - Individual appraisal]

Probe: Why?
Probe: Would your team take part in a study like this again?
[NPT: Reflexive monitoring - Systemization & Communal appraisal]

g. **What did you think of the *[POG/OG]* training?**

Probe: Did it prepare you for using it? If so, how?
Probe: Would you change the training? If so, how?

Part 3. POG/OG Implementation

Now we'll talk about the future use of the POG/OG, by other health professionals and other women with breast cancer. I'd like to know how you think it should be used.

a. **On a scale of 0 to 10, how likely are you to recommend POG/OG to another health professional?**

   Probe: Why?
   Probe: Who would you recommend it to? Why?

b. **Do you think POG/OG should be used in breast cancer care in the future?**

   Probe: Why or why not?
   Probe: On a scale of 0 to 10 how likely are you to use POG/OG now that you're done participating in the study?

   Probe, after giving rating: Why?

   Probe, if rating is over 5: Would it change your practice?
   Probe, if yes: How?

   Probe: For your clinic, what does the ideal process look like for you to use it? Are there any specific actions or procedures that would be needed?
   [NPT: Cognitive participation - activation]

   Probe: Do you have any concerns about using it with your patients?

c. **There are many different ways that women could use POG/OG. In your opinion, how should women receive it?**
   [NPT: Collective Action - Interactional workability]
   [NPT: Collective Action - Skill set workability]

   Let him/her respond then go through the following options if not mentioned:

   Who: Given by the clinician during the visit, as was done during the trial?
   Given by a nurse or other care team member before the visit?
   Is there anyone else women should use it with?

   How: On paper?

Accessible on the patient portal (*can give example for your hospital system, e.g., eD-H, myChart*)?
Included in the after visit summary?
Sent via email?
On a mobile phone?

Should it be included in the electronic health record?

When: When do you think is the best time to use it?
Probe: Before the clinic visit? In the waiting room? At home before the visit?
Probe: During the clinic visit only?
Probe: After the clinic visit?
Probe: Before, during and after?

Where: Where do you think women should use the POG/OG?
In the exam room?
In your office?
Waiting room?
At home, before or after the visit?
[NPT: Cognitive Participation - Legitimation]

d. **Do you have any interest in the tool you did not use in the trial?**
[Can describe and/or offer to email a copy]

Part 4: Additional Relevant Information

Before we finish, I need a little more information about you.

a. **Could you please tell me when you graduated from medical school?**

b. **How long have you been in your current position as a surgeon at [*hospital name*]?**

a. **Could you please summarize your views about shared decision making in a couple of sentences?**

Probe: Did you have an interest in shared decision making before you participated in this trial?
Probe: Has this interest changed now?
Probe, if no: Do you have an interest in shared decision making now?

b. **What were your views of decision aids before this trial?**

Wrap Up

Thank you very much for your time today, and for your participation in the trial. Is there anything else you'd like to share about what we have discussed or about your involvement in the study before we finish up this conversation?

Allow him/her to give any additional feedback.

AIM 3: Explore strategies that promote the encounter decision aids' sustained use and dissemination using a theoretical implementation model.

Hypothesis 1. Pre-visit planning, minimal clinician training, flexibility of use, and integration into the workflow and EMR will facilitate sustained use.

Hypothesis 2. Successful use by patients and their families will be determined by the perceived acceptability of the intervention and integration into workflows.

++

Notes on interview probing

Please always remember that if you are not getting clear answers to your questions, use the following probes (or your own) to get the content and answer to the question you have just asked:

- In what way?
- Could you tell me more?
- Why or why not?

++

Notes on redirection

Here are a few examples on how to redirect the interviewee if s/he begins to go off topic and away from the content of the interview:

- Acknowledge what the participant has said then redirect the conversation.
- Rephrase what the participant said to acknowledge her thoughts then can say "let's take a step back" and then repeat the question.
- Can say thank you for sharing if it feels appropriate.

++

Interview guide begins on the next page.

Introduction

Hi, I'm _____________. Thanks for chatting. Today, we'll talk about your involvement in the Matters Most trial and your experience in the usual care arm. Although I am a part of The Dartmouth Institute, I'm not directly involved in the trial. I hope you'll feel free to be as open in our discussion as possible.

Do you have any questions before we begin?

Review any questions they have.

We'd like to record this conversation so that we can capture everything you say. We'll remove anything that could identify you by name before analysis so nothing you say today can be attributed to you personally. Are you comfortable with me audio recording our conversation?

Based on the answer, turn the recorder on. Check to be sure the recorder is on. Proceed with the interview.

Take detailed notes if they say no to recording.

Part 1. Your Involvement in the Matters Most Trial

a. **Overall, what was it like to take part in the Matters Most study?** *[NPT: Coherence - Individual specification]*

Probe: The goal of the trial was to involve patients in decisions using tools, any comments?
Probe: What was it like for your team?
[NPT: Coherence - Communal specification; Cognitive participation]
Probe: Were there differences from your usual practice?
[NPT: Coherence - Differentiation]
Probe: Was it challenging? If yes, how, and why?
Probe: On a scale of 0 to 10 with 0 meaning no effect, how much did the study affect the clinic workflow?
Probe: How, and why?
Probe: Did you benefit from participating in the study? If yes, how, and why?
[NPT: Coherence - Internalization; Reflexive monitoring]

b. **(if not covered in answers to question a) Did you have to make changes to comply with the study procedures?**
[NPT: Coherence - Differentiation & Collective action]

c. **(If not covered in answers to question a) Did your team have to make changes to comply with the study procedures?**
[NPT: Coherence - Differentiation]

d. **What were your interactions like with the research team?**

Probe: Was it easy or difficult to work with the research team?
Probe: Did you encounter [*name of patient associate*], the patient associate?
Probe: What was it like to work with [*name of patient associate*]?
Probe: In general, what would you change about the involvement of a patient associate?

e. **Could the study team have improved surgeon and team involvement in the study?**
[NPT: Coherence - Reconfiguration]

Probe: If yes, how?

f. **On a scale of 0 to 10 how likely are you to take part in a study like Matters Most again with 0 being least likely and 10 being most likely?**

[NPT: Reflexive monitoring - Individual appraisal]

Probe: Why?
Probe: Would your team take part in a study like this again?
[NPT: Reflexive monitoring - Systemization & Communal appraisal]

g. **What did you think of the trial training?**

Probe: Did it prepare you for the trial? If so, how?
Probe: Would you change the training? If so, how?

Part 2. POG/OG Perception

Now we'll talk about your perception of the tools used in the study. Here are the two tools - Picture Option Grid and Option Grid - that other surgeons in the trial used.

a. **Did you have any awareness of the interventions used in the trial?**
Have both the POG and OG available to show or email to the surgeon.

Probe: Do you have any opinions about these tools?

b. **How did you feel about not using one of the tools in the trial?**

c. **Probe: On a scale of 0 to 10 how likely are you to use the POG or OG now that the trial is over?**

Probe: Why?
Probe: Which one?

On a scale of 0 to 10, how interested are you in using either the Picture Option Grid or Option Grid now?

Probe, after giving rating: Why?

Probe, if rating is over 5: Would it change your practice?
Probe, if yes: How?

Probe, if rating is over 5: What is the ideal scenario for you to use it?
[NPT: Cognitive participation - activation]

Probe: Do you have any concerns about using it with your patients?

d. **Did you hear any of your colleagues talk about the POG/OG over the course of the trial?**

Probe, if yes: Which tool did they talk about?

Probe, if yes: What did they say?

e. **There are many different ways that women could use POG/OG. In your opinion, how should women receive it?**
[NPT: Collective Action - Interactional workability]
[NPT: Collective Action - Skill set workability]

Let him/her respond then go through the following options if not mentioned:

Who: Given by the clinician during the visit, as was done during the trial?
Given by a nurse or other care team member before the visit?
Is there anyone else women should use it with?

How: On paper?
Accessible on the patient portal (*can give example for your hospital system, e.g., eD-H, myChart*)?
Included in the after visit summary?
Sent via email?
On a mobile phone?

Should it be included in the electronic health record?

When: When do you think is the best time to use it?
Probe: Before the clinic visit? In the waiting room? At home before the visit?
Probe: During the clinic visit only?
Probe: After the clinic visit?
Probe: Before, during and after?

Where: Where do you think women should use the POG/OG?
In the exam room?
In your office?
Waiting room?
At home, before or after the visit?
[NPT: Cognitive Participation - Legitimation]

Part 3: Additional Relevant Information

Before we finish, I need a little more information about you.

a. **Could you please tell me when you graduated from medical school?**

b. **How long have you been in your current position as a surgeon at [*hospital name*]?**

a. **Could you please summarize your views about shared decision making in a couple of sentences?**

Probe: Did you have an interest in shared decision making before you participated in this trial?

Probe: Has this interest changed now?
Probe, if no: Do you have an interest in shared decision making now?

b. **What were your views of decision aids before this trial?**

Wrap Up

Thank you very much for your time today, and for your participation in the trial. Is there anything else you'd like to share about what we have discussed or about your involvement in the study before we finish up this conversation?

Allow him/her to give any additional feedback.

Supplying tools?

AIM 3: Explore strategies that promote the encounter decision aids' sustained use and dissemination using a theoretical implementation model.

Hypothesis 1. Pre-visit planning, minimal clinician training, flexibility of use, and integration into the workflow and EMR will facilitate sustained use.

Hypothesis 2. Successful use by patients and their families will be determined by the perceived acceptability of the intervention and integration into workflows.

++

Notes on interview probing

Please always remember that if you are not getting clear answers to your questions, use the following probes (or your own) to get the content and answer to the question you have just asked:

- In what way?
- Could you tell me more?
- Why or why not?

++

Notes on redirection

Here are a few examples on how to redirect the interviewee if s/he begins to go off topic and away from the content of the interview:

- Acknowledge what the participant has said then redirect the conversation.
- Rephrase what the participant said to acknowledge her thoughts then can say "let's take a step back" and then repeat the question.
- Can say thank you for sharing if it feels appropriate.

++

Interview guide begins on the next page.

Introduction

Hi, I'm _____________. Thanks for chatting. Today, we'll talk about your involvement in the Matters Most trial (if applicable). Although I am a part of The Dartmouth Institute, I'm not directly involved in the trial. I hope you'll feel free to be as open in our discussion as possible.

Do you have any questions before we begin?

Review any questions they have.

We'd like to record this conversation so that we can capture everything you say. We'll remove anything that could identify you by name before analysis so nothing you say today can be attributed to you personally. Are you comfortable with me audio recording our conversation?

Based on the answer, turn the recorder on. Check to be sure the recorder is on. Proceed with the interview.

Take detailed notes if they say no to recording.

Part 1. Your Involvement in the Matters Most Trial

a. **Can you tell me about what you know about the What Matters Most study?**

Probe: The goal of the trial was to involve patients in decisions using tools, any comments?

b. **Can you tell me about your involvement in the Matters Most study?**

Probe: What specific things did you do for the study?

c. **What was it like to take part in the Matters Most study?** *[NPT: Coherence - Individual specification]*

Probe: What was it like for your team?
[NPT: Coherence - Communal specification; Cognitive participation]
Probe: How was this different from your usual practice?
[NPT: Coherence - Differentiation]
Probe: How easy or challenging was it to take part in the study?
Probe if clinical team member: On a scale of 0 to 10 with 0 meaning no effect, how much did the study affect the clinic workflow?
Probe: How, and why?
Probe: Did you notice if it changed the length of the consultation?
Probe: Did you benefit from participating in the study? How? And why or why not?
[NPT: Coherence - Internalization; Reflexive monitoring]

d. **(if not covered in answers to question a) Did you have to make changes to comply with the study procedures?**
[NPT: Coherence - Differentiation & Collective action]

e. **(If not covered in answers to question a) Did your team have to make changes to comply with the study procedures?**
[NPT: Coherence - Differentiation]

f. **What were your interactions like with the research team?**

Probe: Was it easy or difficult to work with the research team?
Probe: Did you encounter [*name of patient associate*], the patient associate?
Probe: What was it like to work with [*name of patient associate*]?

Probe: In general, what would you change about the involvement of a patient associate?

g. **Could the study team have improved team/your involvement in the study?** *[NPT: Coherence - Reconfiguration]*

Probe: If yes, how?

h. **On a scale of 0 to 10 how likely are you to take part in a study like Matters Most again with 0 being least likely and 10 being most likely?**
[NPT: Reflexive monitoring - Individual appraisal]

Probe: Why?
Probe: If applicable, would your team take part in a study like this again?
[NPT: Reflexive monitoring - Systemization & Communal appraisal]

i. **Were you trained for your involvement in the trial? What did you think of the training?**

Probe: Did it prepare you for the trial? If so, how?
Probe: Would you change the training? If so, how?

Part 2. POG/OG Perception

Now we'll talk about your perception of the tools used in the study. Here are the two tools - Picture Option Grid and Option Grid - that clinicians used in the trial.

a. **Did you have any awareness of the interventions used in the trial?**
Have both the POG and OG available to show or email to the interviewee.

Probe: Do you have any opinions about these tools?

b. **On a scale of 0 to 10 how likely are you to support continued use of the POG or OG now that the trial is over?**

Probe: Why?
Probe: Which one?
Probe: Would you recommend these tools to others?

c. **Did you hear any of your colleagues talk about the POG/OG over the course of the trial?**

Probe, if yes: Which tool did they talk about?

Probe, if yes: What did they say?

d. Probe: What was it like to integrate the intervention into your usual way of working?
[NPT: Cognitive participation; Reflexive monitoring]

e. **There are many different ways that women could use POG/OG. In your opinion, how should women receive it?**
[NPT: Collective Action - Interactional workability]
[NPT: Collective Action - Skill set workability]

Let him/her respond then go through the following options if not mentioned:

Who: Given by the clinician during the visit, as was done during the trial?
Given by a nurse or other care team member before the visit?
Is there anyone else women should use it with?

How: On paper?
Accessible on the patient portal (*can give example for your hospital system, e.g., eD-H, myChart*)?
Included in the after visit summary?
Sent via email?
On a mobile phone?

Should it be included in the electronic health record?

When: When do you think is the best time to use it?
Probe: Before the clinic visit? In the waiting room? At home before the visit?
Probe: During the clinic visit only?
Probe: After the clinic visit?
Probe: Before, during and after?

Where: Where do you think women should use the POG/OG?
In the exam room?
In your office?
Waiting room?
At home, before or after the visit?
[NPT: Cognitive Participation - Legitimation]

Part 3: Additional Relevant Information

Before we finish, I need a little more information about you.

a. **What are your current position(s) and responsibilities at [*hospital name*]?**

b. **How long have you been in your current position at [*hospital name*]?**

c. **Could you please summarize your views about shared decision making in a couple of sentences?**

   Probe: Did you have an interest in shared decision making before you participated in this trial?

      Probe: Has this interest changed now?
      Probe, if no: Do you have an interest in shared decision making now?

d. **What were your views of decision aids before this trial?**

Wrap Up

Thank you very much for your time today, and for your support during the study. Is there anything else you'd like to share about what we have discussed or about your involvement in the study before we finish up this conversation?

Allow him/her to give any additional feedback.

Supplying tools?

AIM 3: Explore strategies that promote the encounter decision aids' sustained use and dissemination using a theoretical implementation model.

Hypothesis 1. Pre-visit planning, minimal clinician training, flexibility of use, and integration into the workflow and EMR will facilitate sustained use.

Hypothesis 2. Successful use by patients and their families will be determined by the perceived acceptability of the intervention and integration into workflows.

++

Notes on interview probing

Please always remember that if you are not getting clear answers to your questions, use the following probes (or your own) to get the content and answer to the question you have just asked:

- In what way?
- Could you tell me more?
- Why or why not?

++

Notes on redirection

Here are a few examples on how to redirect the interviewee if s/he begins to go off topic and away from the content of the interview:

- Acknowledge what the participant has said then redirect the conversation.
- Rephrase what the participant said to acknowledge her thoughts then can say "let's take a step back" and then repeat the question.
- Can say thank you for sharing if it feels appropriate.

++

Interview guide begins on the next page.

Aim 3 - Non-Clinical Stakeholder Interview Guide

Have both the POG and OG available to show or email to the interviewee.

Introduction

Hi, I'm ____________. Thanks for chatting. Today, we'll talk about the What Matters Most trial of breast cancer patient decision aids. Although I am a part of The Dartmouth Institute, I'm not directly involved in the trial. I hope you'll feel free to be as open in our discussion as possible.

The information sheet Kate emailed to you earlier is primarily intended to let you know that 1) today's discussion is for research purposes; 2) participation is voluntary and you can skip any questions that you'd like; and 3) the information you discuss will be kept confidential won't be attributed to you by name in any reports or presentations about this project.

Do you have any questions before we begin?

Review any questions they have.

We'd like to record this conversation so that we can capture everything you say. We'll remove anything that could identify you by name before analysis so nothing you say today can be attributed to you personally. Are you comfortable with me audio recording our conversation?

Based on the answer, turn the recorder on. Check to be sure the recorder is on. Proceed with the interview.

Take detailed notes if they say no to recording.

WHAT MATTERS MOST

Part 1. The Matters Most Trial

a. **Do you know about the What Matters Most study?**

If yes, probe: Could you tell me what you know about the study?

If no, explain: It is a multi-site randomized controlled trial that aims to help women newly diagnosed with breast cancer decide which surgical option is best for them. The research team evaluated two encounter patient decision aids against usual care. One encounter patient decision aid uses pictures and simple words to help women decide between mastectomy and lumpectomy with radiation. The other encounter patient decision aid uses the same tabular format but does not have pictures and fits on one page. You should have received a PDF copy of these tools. They used paper versions in the trial.

For both: Do you have any comments or questions about the study?

b. **Did you have any involvement in the Matters Most study?**

Probe: What specific things did you do for the study?

c. **Did you hear or speak with anyone about the study while the research team was recruiting patient participants?**

Probe, if yes: What did hear or what did you speak about?

d. **Did you have any interactions with the research team?**

Probe, if yes: What were these interactions?

Probe: Was it easy or difficult to work with the research team?

e. **In this study, the research team involved breast cancer survivors, which they called patient associates, as study team members involved in recruitment, consent and data collection. What are your thoughts on this role?**

Probe: What concerns, if any, do you have?

f. **On a scale of 0 to 10 how likely are you to support a study like Matters Most with 0 being least likely and 10 being most likely?**

[NPT: Reflexive monitoring - Individual appraisal]

Probe: Why?
Probe: If applicable, would your team or institution take part in a study like this?
[NPT: Reflexive monitoring - Systemization & Communal appraisal]

Part 2. POG/OG Perception

Now we'll talk about your perception of the tools used in the study. As mentioned earlier you have received copies of the two tools - Picture Option Grid and Option Grid - that clinicians used in the trial. Do you have them in front of you?

a. **What is your impression of the tools?**

Probe: What stands out?
Probe: What do you like?
Probe: What do you dislike?
Probe: What could be improved?
Probe: Do you have a preference between the two tools?

b. **On a scale of 0 to 10 how likely are you to support continued use of the POG or OG outside of the Matters Most trial?**

Probe: Why?
Probe: Which one?
Probe: Would you recommend these tools to others?

c. **Did you hear any of your colleagues talk about the POG or OG over the course of the trial?**

Probe, if yes: Which tool did they talk about?
Probe, if yes: What did they say?

For EHR executives/interviewees

d. **What concerns, if any, do you have about the integration of the interventions into the electronic health record?**

Probe: Have you done this before?
Probe: What existing support is there to integrate these sorts of tools into the EHR?

Probe: What would be required to integrate a link to the existing online version of the text Option Grid?

e. **The online text Option Grid uses single sign on, do you have any concern about single sign on for integration of this tool?**

f. **What would be required to integrate a PDF of the Picture Option Grid into EPIC?**

For higher level stakeholders (e.g., executive directors, etc.)

g. **What concerns, if any, do you have about the integration of the interventions into the clinic workflow?**

h. **There are many different ways that women with breast cancer could use POG/OG. In your opinion, how should women receive it?**
[NPT: Collective Action - Interactional workability]
[NPT: Collective Action - Skill set workability]

Part 3: Additional Relevant Information

Before we finish, I need a little more information about you.

a. **What are your current position(s) and responsibilities at [*hospital name*]?**

b. **How long have you been in your current position at [*hospital name*]?**

c. **Could you please summarize your views about shared decision making in a couple of sentences?**

Probe: Did you have an interest in shared decision making before your cancer center participated in this trial?

Probe: Has this interest changed now?
Probe, if no: Do you have an interest in shared decision making now?

Wrap Up

Thank you very much for your time today. Is there anything else you'd like to share about what we have discussed before we finish up this conversation?

Allow him/her to give any additional feedback.
