# Supplementary material for: Implementation and sustainability factors of two early-stage breast cancer conversation aids in diverse practices
Source: Implement Sci. 2021 May 10;16:51. doi: 10.1186/s13012-021-01115-1 (PMC8108365; doi:10.1186/s13012-021-01115-1)
Supplement: Supplementary file 6 — Additional file 6. [file 13012_2021_1115_MOESM6_ESM.pdf]

WHAT MATTERS MOST

Observation Grid

Name ______________________

Date ______________________

Time ______________________

Site ______________________

	Probes	Waiting Rooms	Hallways/Staff Areas
MATERIALALS (Observe)	Visible materials e.g., pamphlets, books		
	Where are the materials? How are they presented?		
	Who is interacting with the materials?		
	Any modification in the use of Option Grid or Picture Option Grid?		
	Are the clinicians using the tools according to the protocol?		
CHATTER (Observe)	Are clinicians/ patients discussing the materials? What are they saying?		
	What questions are patients asking about the materials?		
	Any expression of confusion, frustration, relief, etc.?		
	Are the clinicians using the tools according to the protocol?		

Observation Grid

WHAT MATTERS MOST

Name ____________________

Date ____________________

Time ____________________

Site ____________________

QUESTIONS (Ask)			
	What do you think of that document?		
	Have you had to replace the materials at all?		
	How is it going with the WMM materials?		
	How did that consultation go?		
	Is anything on your mind?		
FIELD NOTES (Reflection)			
