# Supplementary material for: Implementation and sustainability factors of two early-stage breast cancer conversation aids in diverse practices
Source: Implement Sci. 2021 May 10;16:51. doi: 10.1186/s13012-021-01115-1 (PMC8108365; doi:10.1186/s13012-021-01115-1)
Supplement: Supplementary file 7 — Additional file 7. [file 13012_2021_1115_MOESM7_ESM.pdf]

NOMAD ITEMS BY CONSTRUCT

Construct	Sub-Construct	Items
Coherence	***Differentiation***	I can see how the [intervention] differs from usual ways of working
	Communal specification	Staff in this organisation have a shared understanding of the purpose of this [intervention]
	Individual specification	I understand how the [intervention] affects the nature of my own work
	Internalization	I can see the potential value of the [intervention] for my work
Cognitive Participation	***Initiation***	There are key people who drive the [intervention] forward and get others involved
	Legitimation	I believe that participating in the [intervention] is a legitimate part of my role
	Enrolment	I'm open to working with colleagues in new ways to use the [intervention]
	Activation	I will continue to support the [intervention]
Collective Action	***Interactional workability***	I can easily integrate the [intervention] into my existing work
	Relational integration	The [intervention] disrupts working relationships
	Relational integration	I have confidence in other people's ability to use the [intervention]
	Skill set workability	Work is assigned to those with skills appropriate to the [intervention]
	Skill set workability	Sufficient training is provided to enable staff to use the [intervention]
	Contextual Integration	Sufficient resources are available to support the [intervention]
	Contextual integration	Management adequately support the [intervention]
Reflexive Monitoring	***Systemisation***	I am aware of reports about the effects of the [intervention]
	Communal appraisal	The staff agree that the [intervention] is worthwhile
	Individual appraisal	I value the effects the [intervention] has had on my work
	Reconfiguration	Feedback about the [intervention] can be used to improve it in the future
	Reconfiguration	I can modify how I work with the [intervention]
