# Supplementary material for: Implementation and sustainability factors of two early-stage breast cancer conversation aids in diverse practices
Source: Implement Sci. 2021 May 10;16:51. doi: 10.1186/s13012-021-01115-1 (PMC8108365; doi:10.1186/s13012-021-01115-1)
Supplement: Supplementary file 8 — Additional file 8. [file 13012_2021_1115_MOESM8_ESM.docx]

**Appendix 8. Average length of interviews stratified by site and interviewee type**

| **Interviewee** | **Average length**  **(in minutes)** |
| --- | --- |
| **Patients** | **24** |
| Site 1 | 30 |
| Site 2 | 16 |
| Site 3 | 21 |
| Site 4 | 28 |
| **Surgeons** | **27** |
| Site 1* | 27 |
| Site 2 | 25 |
| Site 3 | 30 |
| Site 4 | 25 |
| **Stakeholders** | **20** |
| Site 1** | 23 |
| Site 2* | 19 |
| Site 3 | 14 |
| Site 4 | 24 |
| *1 no recording, notes taken  **2 no recording, notes taken | |
