# Supplementary material for: Implementation and sustainability factors of two early-stage breast cancer conversation aids in diverse practices
Source: Implement Sci. 2021 May 10;16:51. doi: 10.1186/s13012-021-01115-1 (PMC8108365; doi:10.1186/s13012-021-01115-1)
Supplement: Supplementary file 9 — Additional file 9. [file 13012_2021_1115_MOESM9_ESM.docx]

**Appendix 9. Major and minor themes by NPT construct**

| **Major and minor themes** | **Quotations** |
| --- | --- |
| **Construct: Coherence - What is the work?** | |
| *Differentiation* | |
| **Patients**  Major: Regardless of SES and intervention received, most patients felt the conversation aids were easier to understand and were more concise compared to other materials they had received (39/42). | “*It seemed more specific and simple. Easy to read and understand.*” - Patient, POG, Higher SES  "*I just thought it made it easier to understand, and then it helped with making the decision that was best for me*." - Patient, OG, Lower SES |
| **Patients**  Minor: Twelve patients felt the conversation aids were not different than materials they have received in the past. | "*It pretty much followed along with the other information that we’ve been probed or read about.*" - Patient, OG, Higher SES |
| **Surgeons**  Major: For most intervention surgeons, the use of the conversation aids felt similar (8/11) to their usual care. | "*It’s not too different from my normal practice and so it was pretty straightforward.*" - Surgeon, POG |
| *Communal specification* - No major or minor themes associated with this component. | |
| *Individual specification* | |
| **Patients**  Major: Regardless of SES and intervention received, most patients understood that the conversation aids were being used with them in the appointment to help them compare their treatment options and make a decision aligned with their preferences (38/42). Some patients also understood that the tool served as a starting point to their conversation with their surgeon (13/42). | "*To let me know that it’s my choice to be clear about what my options were and outcomes and risk factors and just everything, to get me involved with the decision-making.*" - Patient, POG, Higher SES  "*And how did I use it? I used it as a starting point in exploring how to approach this disease and what to do with it, how, surgically what to do.*" - Patient, POG, Higher SES |
| **Patients**  Minor: Regardless of SES and intervention, all patients found the conversation aids easy to understand (42/42). | "*Because it’s easy to read. I know with so much family and friends may not have the understanding that I do with medical things, and this is easy to read and understand.*" - Patient, OG, Higher SES |
| **Surgeons**  Minor: Five out of 11 intervention surgeons mentioned that they felt the conversation aids helped compare treatment options. | "*I think that it helped to click to understand what their two options were. I do feel like sometimes when I explain the two options without using the Picture Option Grid, it’s a little bit more difficult for them to grasp. My hope would be that it does make the decision-making easier for them*." - Surgeon, POG |
| *Internalization* | |
| **Patients**  Major: Regardless of SES, most patients liked the conversation aids they received (34/42). | "*I like the fact that it’s just a one-page thing. I mean a lot of the stuff that you can find about breast cancer might go on and on and, of course, everybody’s situation is a little bit different. So, this is kind of consolidating the two biggest options, if you will, into a really easy to read tool*." - Patient, OG, Higher SES |
| **Patients**  Minor: Regardless of SES and intervention received, almost all patients took the tool home (34/42); half said the tool reassured them about their treatment choice or was used for further deliberation (24/42), and half used it with other individuals like friends and family (12/24). | "*That was it then with my – I then took the chart and I reviewed when I was home and then decided on what I wanted to do*." - Patient, POG, Higher SES |
| **Surgeons and stakeholders**  Major: Regardless of intervention used, over half of intervention surgeons (6/11) and three stakeholders felt that the conversation aids would be helpful for patients with lower health literacy. | "*When you're sitting there and actually showing them the picture and they can visualize what you're telling them, it does help, especially in our patient population where they don't necessarily understand everything we are saying to them*." - Clinical stakeholder  "*I can definitely see there are differences in socioeconomic or different educational backgrounds where actual pictures might be much more powerful.*" - Surgeon, POG |
| **Construct: Cognitive participation - Who does the work?** | |
| *Initiation* | |
| **Stakeholders**  Minor: One trial site out of four had medical assistants who would punch holes in the conversation aids and insert them in the surgeon’s usual care binder. | "*The Option Grids continue to be incorporated into the binders that Dr. X provides new cancer patients.*" - Clinical stakeholder |
| *Enrollment* | |
| **Patients**  Major: Regardless of SES, almost all patients highlighted the importance of paper-based materials (40/42). However, about half of patients, regardless of SES and intervention received, were also open to other methods of receiving the conversation aids including email, mail, or a patient portal.  Some patients, regardless of SES, were more likely to indicate that receiving the conversation aid from their surgeon is best (30/42). | "*Being someone who does not have any access to computers and all the rest, it’s much easier for me to have a hard copy. I can take it home with me.*" Patient, OG, Lower SES  "*I think if it had been emailed to me before, maybe it might have helped me, to have it before my office visit, process the information.*" - Patient, POG, Higher SES  "*I liked having it with the doctor*." - Patient, OG, Lower SES |
| **Patients**  Minor: Provided patients knew their diagnosis ahead of the surgical consultation, some patients said they would want to receive the conversation aids before their appointment so they could review it and have more directed questions (15/42). | "*I would give it to me on paper but I would also send it by email, [patient portal name], or – it doesn’t have to be one way. It could be two ways, it could be three ways.*" Patient, POG, Higher SES |
| **Surgeons**  Major: Half of the participating surgeons indicated that patients receiving the conversation aids before their appointment would be helpful (8/16). | "*I think to me, it seems like having something beforehand, even if it’s just a heads-up, would be helpful.*" - Surgeon, OG |
| **Surgeons**  Minor: Surgeons felt that using the tool with their patients together is best, particularly in paper format. Regardless of intervention used, there were mixed feelings about EHR integration: 11 thought it would be helpful and six did not. However, regardless of intervention used, some intervention surgeons favored integrating the conversation aids into the patient portal (7/11). | "*I think the paper format; you print it out and give it to the patients while you’re going through it with them so they can be holding on to it. I think that’s helpful. And then if you wanted to have an electronic format, just maybe like on the cancer center’s website or something that you could access if they lost their sheet, that would be fine.*" - Surgeon, POG  "*I think if there was a shared access with patients and point questions, there could be ways that there’s some sort of interactive grid that we could communicate via [patient portal system]. That potentially could be beneficial.*" - Surgeon, OG |
| *Legitimation* | |
| **Patients**  Major: Regardless of SES and intervention received, almost all patients recommended that others should use the conversation aids (40/42). | "*I will definitely recommend it. I think it was a great piece of paper. I’d let them read my notes, I’d help them in any way possible, but I thought it was a great piece of paper to give you comfort, to give you enough information, to give you comfort that it’s all going to be okay.*" - Patient, POG, Higher SES  "*I would recommend it to everybody.*" - Patient, OG, Lower SES |
| **Surgeons**  Major: Most surgeons recommended that other health professionals use the conversation aids (14/16). | (after recommending the tool) "*Well, I think it could be used by anybody who sees patients with breast cancer who are making these decisions.*" - Surgeon, POG |
| *Activation* - No major or minor themes associated with this component. | |
| **Construct: Collective action - How does the work get done?** | |
| *Interactional workability* | |
| **Patients**  Major: Regardless of SES and intervention received, over half of patients felt their surgeons used the conversation aids with ease and believed they were a part of normal routine (29/42). | “*It flowed into our appointment so seamlessly. It definitely seemed like part of how she would present the information.*" - Patient, POG, Higher SES |
| **Patients**  Minor: Regardless of intervention received, most patients were satisfied with when the conversation aids were used with them during the appointment (34/42). The conversation aids were used at varying times depending on site and surgeon. | "*Yes, I would think [right time]. I mean you want to know what your diagnosis is and then once you fully understand that, you talk about what you're going to do about it.*" - Patient, OG, Higher SES |
| **Surgeons**  Major: Regardless of intervention used, most intervention surgeons felt they were able to integrate the conversation aid as part of their normal routine (8/11). Over half of the intervention surgeons noted that it took using the tool a few times before they could use it with ease (7/11). | "*Well, it became pretty much second-hand for me actually. I got really used to using it so I don’t know. It just became part of my routine.*" - Surgeon, POG  “*The more I used it, it just became more part of my practice. I would go through it more seamlessly and quicker as time went on.*” - Surgeon, OG |
| **Surgeons**  Minor: Regardless of intervention used, for a minority of intervention surgeons, the conversation aids felt awkward and time-consuming, and they preferred their usual method of delivering information (3/11). | "*Like I said, it was awkward. It didn’t flow with the conversation that I usually have.*" - Surgeon, POG |
| *Relational integration* - No major or minor themes associated with this component. | |
| *Skillset workability* | |
| **Surgeons**  Major: Regardless of intervention used, all surgeons thought the training on using the tool, or usual care, was sufficient, easy, and did not suggest any changes (16/16). | "*I thought it was good. I thought it was very easy how they just kind of roleplay a little bit to work it out but I thought it went very smoothly. [...] I don’t think so [change anything about training]. I think it was great how they went through the steps and then the roleplaying of course but, no, I don’t think I would change that.*" - Surgeon, POG |
| *Contextual integration* - No major or minor themes associated with this component. | |
| **Reflexive monitoring - How is the work understood?** | |
| *Systemization* | |
| **Patients**  Major: Regardless of intervention received, over half of the patients felt that the tool affected their treatment decision (30/42). Regardless of SES and intervention received, a little under half of patients felt the tool helped them understand their options. Sixteen patients reported an increased understanding due to the conversation aids. | "*I had a lumpectomy instead of a mastectomy because the information contained in the grid helped me understand that – not that I ever thought I wanted a mastectomy but it helped me understand that I didn’t need one.*" - Patient, POG, Higher SES  "*It did help me decide which one I wanted because it told me what a lumpectomy was and what a mastectomy was, the difference.*" Patient, OG, Higher SES |
| *Communal appraisal* - No major or minor themes associated with this component. | |
| *Individual appraisal* | |
| **Surgeons**  Major: For most intervention surgeons, both conversation aids were understood to help structure the conversation about making a treatment decision (8/11). | "*It did help the rhythm. At first, it took me a little bit longer than I think it would’ve been, but not much. Then at the end, I think it ultimately helped structure things. It might have made things as efficient or more efficient.*" - Surgeon, OG |
| *Reconfiguration* | |
| **Patients**  Major: Sixteen patients reported that their surgeons customized the conversation aids by writing and drawing on the conversation aid. | "*I believe she turned the paper over and made a sketch on it to show me something that she was talking about.*" - Patient, OG, Higher SES |
| *Unspecified* | |
| **Patients**  Major: Most patients felt that the conversation aids should be used in the future (35/42). | "*I would say, as somebody who’s been through it, I would use this definitely for any future breast cancer people because it’s pretty black and white and it answers.*" - Patient, OG, Lower SES |
