# Supplementary material for: Implementation and sustainability factors of two early-stage breast cancer conversation aids in diverse practices
Source: Implement Sci. 2021 May 10;16:51. doi: 10.1186/s13012-021-01115-1 (PMC8108365; doi:10.1186/s13012-021-01115-1)
Supplement: Supplementary file 10 — Additional file 10. [file 13012_2021_1115_MOESM10_ESM.docx]

**Appendix 10. Major and minor themes by participant SES**

| **Construct themes** | **Quotations** |
| --- | --- |
| **Coherence - What is the work?** | |
| Patients of higher SES (6/30) were more likely to mention that the conversation aids were plain language than patients of lower SES (0/12). | "*Because it was so clear. It was so clear. It was so easy to read and understand.*" - Patient, POG, Higher SES |
| Almost half of the patients of higher SES mentioned that the conversation aids were concise (15/30) compared to a third of patients of lower SES (4/12). | "*I thought it was very good, concise, and easy to understand.*" - Patient, OG, Higher SES |
| All patients of higher SES understood that the conversation aids were meant to compare their treatment options (30/30) compared to only about half of patients of lower SES (7/12). | "*I think to let me know that I had choices and what the percentages were – like what the outcome and choices are, and then also to explain better what the process is between lumpectomy and radiation. Before I went into the office, I didn’t understand the differences between the outcomes fully. By the time I exited the office, I felt like I had a much better vision of what that looked like.*" - Patient, POG, Higher SES |
| Patients of higher SES were more likely to mention, not prompted, that the conversation aids served as a starting point for a conversation with their surgeon (11/30) compared to patients of lower SES (2/12). | "*It was just basically a tool to use to help trigger conversation points during that meeting.*" - Patient, OG, Higher SES |
| Most patients of higher SES mentioned that the conversation aids were easy to use (29/30) compared to only about half of patients of lower SES (7/12). | "*It did such a good job of showing the two options and especially the visual of what the surgery would look like and what radiation looks like and what chemotherapy looks like. That’s why I would absolutely point somebody in the direction of this tool.*" - Patient, POG, Higher SES |
| Patients of higher SES were more likely to use the conversation aids at home (19/30) compared to patients of lower SES (5/12), and more than half of the patients of higher SES who used the conversation aids at home did so for reassurance (10/19) compared to patients of lower SES (2/5). | “*I think it was to reassure me of what - it means that I had questions in between a lumpectomy and a mastectomy, and I think it was to reassure me about many things*.” - Patient, OG, Higher SES |
| **Cognitive participation - Who does the work?** | |
| Patients of higher SES were more likely to indicate that receiving the tool ahead of their appointment would be helpful (13/30) compared to patients of lower SES (2/12). | "*It would be helpful for me to have it at home before the appointment.*" - Patient, OG, Higher SES |
| Patients of higher SES were more likely to mention that they would prefer to have the tool mailed to their home before the appointment (14/30) compared to patients of lower SES (4/12). | "*Actually, I suppose it could have been actually mailed to you even right after a biopsy or something, so that patients could read it over and try to make a decision.*" - Patient, OG, Higher SES |
| More patients of higher SES (23/30) mentioned that paper-based versions of the conversation aids were important compared to patients of lower SES (6/12). However, patients of higher SES were also more likely to mention that they were comfortable receiving the tool via a patient portal (17/30) compared to patients of lower SES (4/12). | "*You get overwhelmed with a lot of information and having something that you can touch and see – I interacted with the piece of paper a hundred times more than I would with the downloaded file if somebody had just sent that to me to look at before my appointment.*" - Patient, OG, Higher SES |
| **Collective action - How does the work get done?** | |
| Patients of lower SES were more likely to mention that they felt their surgeon was comfortable using the tool with them (10/12) compared to patients of higher SES (17/30). | “[the surgeon] *was very comfortable*.” - Patient, OG, Lower SES |
| More patients of higher SES were more likely to mention that the tool was used with them at the right time (26/30) compared to patients of lower SES (8/12). | "*Yes, I felt that it was the right time to get the tool since they have helpful information on it. Then after what we talked about, having that, it was helpful for me.*" - Patient, POG, Higher SES |
| **Reflexive monitoring - How is the work understood?** | |
| More than half of patients of higher SES felt that the tool affected their discussion with their surgeon (16/30) compared to patients of lower SES (2/12). | "*I think it really influenced our discussion by keeping us on track and talking about the risks/benefits of both options.*" - Patient, OG, Higher SES |
| More patients of lower SES felt that the tool influenced their treatment decision (9/12) compared with patients of higher SES (17/30). | "*I’d say it really influenced it* [my decision]." - Patient, OG, Lower SES |
