# Supplementary material for: Implementation and sustainability factors of two early-stage breast cancer conversation aids in diverse practices
Source: Implement Sci. 2021 May 10;16:51. doi: 10.1186/s13012-021-01115-1 (PMC8108365; doi:10.1186/s13012-021-01115-1)
Supplement: Supplementary file 11 — Additional file 11. [file 13012_2021_1115_MOESM11_ESM.docx]

**Appendix 11. Analysis comparing Picture Option Grid higher SES versus lower SES**

| **Construct themes** | **Quotations** |
| --- | --- |
| **Coherence - What is the work?** | |
| *Differences* | |
| All patients of higher SES who received the Picture Option Grid (18/18) felt that the tool was easier to understand compared to materials they may have received before versus only half of patients of lower SES (3/6). | “*It was easier to understand*.” - Patient, POG, Higher SES |
| Almost all patients of higher SES (17/18) who received the Picture Option Grid mentioned that the tool was easy to understand and easy to use. However, only about half of patients of lower SES (4/6) who received the Picture Option Grid mentioned that the tool was easy to understand and only half (3/6) mentioned that the tool was also easy to use. Of those of lower SES who said the tool was easy to understand, all had mentioned that the images played a significant role in their understanding (4/4). | “*It was very clear and easy to understand.*” - Patient, POG, Higher SES  “*She said the pictures helped her understand more easily*.” - (Interpreter for) Patient, POG, Lower SES |
| Patients of higher SES who received the Picture Option Grid were more likely to use the tool at home for reassurance (5/18) and with others (8/13) compared to patients of lower SES who received the Picture Option Grid (1/6 and 1/2 respectively). | "*We took it out [at home] to show my mother. She came to kind of help take care of me during the process and you know, she said kind of helped her understand the surgery and everything as well.*" - Patient, POG, Higher SES |
| *No differences* | |
| Patients of higher SES who received the Picture Option Grid were about equally as likely to mention that the tool was concise (7/18) than patients of lower SES who received the Picture Option Grid (3/6). | "*It was very easy to understand. It was very clear cut and it wasn’t – it doesn’t have the overwhelming quantity of information that other *laughter* documents have.*" - Patient, POG, Higher SES |
| Patients of higher SES who received the Picture Option Grid were about equally likely to mention that the tool helped them compare their options (16/18) than patients of lower SES who received the Picture Option Grid (4/6). | "*It did such a good job of showing the two options and especially the visual of what the surgery would look like and what radiation looks like and what chemotherapy looks like. That’s why I would absolutely point somebody in the direction of this tool.*" - Patient, POG, Higher SES |
| **Cognitive participation - Who does the work?** | |
| *Differences* | |
| Patients of higher SES who received the Picture Option Grid were more likely to indicate they would like to receive the tool ahead of their appointment with their surgeon (11/18) compared to patients of lower SES who received the Picture Option Grid (1/6), with patients of higher SES suggesting mailing the tool to the patient's home before the appointment (9/11). | "*I think the sooner [to receive the Picture Option Grid], the better.*" - Patient, POG, Higher SES  "*If everyone got this in the mail that would – I think that would have been really helpful.*" - Patient, POG, Higher SES |
| Proportionally, patients of lower SES who received the Picture Option Grid were more likely to indicate that they prefer to receive the tool from their surgeon (5/6) compared to patients of higher SES who received the Picture Option Grid (11/18). | "*She says that it’s better if the doctor gave it to her.*" - (Interpreter for) Patient, POG, Lower SES |
| Over half of patients of higher SES who received the Picture Option Grid mentioned that they prefer to receive the tool in paper format (11/18) compared to patients of lower SES who received Picture Option Grid where almost all mentioned paper-based as their preference (5/6). | "*I would go for the paper option. I know everybody likes to do everything on their phone but I’m still for the paper option.*" - Patient, POG, Lower SES |
| *No differences* | |
| All patients of lower SES who received the Picture Option Grid mentioned that they recommend the tool for others to use (6/6) compared to almost all patients of higher SES who received the Picture Option Grid (14/18). | "*I will definitely recommend it. ...I think it was a great piece of paper. I’d let them read my notes, I’d help them in any way possible, but I thought it was a great piece of paper to give you comfort, to give you enough information, to give you comfort that it’s all going to be okay.*" - Patient, POG, Higher SES  "*Because it gives them the difference between the lumpectomy and the mastectomy, and I think it would be a helpful tool for them to use and to go over with their doctor, so their doctor can help them with that decision..., whether they need to check the lumpectomy or to remove the whole breast or whatever the case may be, or if it’s just a mastectomy. Like, 'Let’s go in and take this piece out and get it tested.' I feel like yes, I would most definitely recommend it.*"- Patient, POG, Lower SES |
| **Collective action - How does the work get done?** | |
| *No differences* | |
| Patients of higher SES who received the Picture Option Grid were almost equally likely to mention that they felt their surgeon was comfortable using the tool with them (16/18) compared to patients of lower SES (4/6). | "*I would assume so because it flowed into our appointment so seamlessly. It definitely seemed like part of how she would present the information.*" - Patient, OG, Higher SES |
| Patients of higher SES who received the Picture Option Grid were almost equally likely to mention that the tool was used with them at the right time (15/18) compared to patients of lower SES (4/6). | "*No, it was perfect. The whole appointment was obviously overwhelming with the new diagnosis and it just flowed properly.*" - Patient, POG, Higher SES |
| Patients of higher SES (15/18) were almost equally likely to take the tool home with them after their appointment with their surgeon than patients of lower SES (4/6). | “*I then took it, and I reviewed when I was home and then decided on what I wanted to do.*” - Patient, POG. Higher SES |
| Patients of lower SES (4/6) and higher SES (12/18) who received the Picture Option Grid equally felt that using the tool did not feel awkward. | "*Never [awkward].*" - Patient, POG, Higher SES |
| Patients of lower SES (4/6) and higher SES (13/18) almost equally felt that the tool was a part of their surgeon's normal routine. | "*Yes, it did [feel like their normal routine].*" - Patient, POG, Higher SES |
| **Reflexive monitoring - How is the work understood?** | |
| *Differences* | |
| Half of patients of higher SES who received the Picture Option Grid felt that the tool influenced their discussion with their surgeon (9/18) compared to patients of lower SES (1/6). | "*Well, I think it put the icing on the cake, how is that – and made it known that the results were about the same and thus said, it was much easier to have a lumpectomy than a mastectomy.*" - Patient, POG, Higher SES |
| Patients of higher SES who received the Picture Option Grid were more likely to mention that the tool affected their treatment decision (15/18) than patients of lower SES (4/6). | "*100% [affected my decision]*"- Patient, POG, Lower SES |
| One third of patients of higher SES who received the Picture Option Grid mentioned that the tool helped them understand their options (6/18) compared to about half of patients of lower SES (4/6). | "*I had a lumpectomy instead of a mastectomy because the information contained in the grid helped me understand that – not that I ever thought I wanted a mastectomy but it helped me understand that I didn’t need one.*" - Patient, POG, Higher SES |
