# Supplementary material for: Implementation and sustainability factors of two early-stage breast cancer conversation aids in diverse practices
Source: Implement Sci. 2021 May 10;16:51. doi: 10.1186/s13012-021-01115-1 (PMC8108365; doi:10.1186/s13012-021-01115-1)
Supplement: Supplementary file 12 — Additional file 12. [file 13012_2021_1115_MOESM12_ESM.docx]

**Appendix 12. Analysis comparing Option Grid higher SES versus lower SES**

| **Construct themes** | **Quotations** |
| --- | --- |
| **Coherence - What is the work?** | |
| *Differences* | |
| Patients of higher SES who received the Option Grid were more likely to mention that the tool was concise (8/12) than patients of lower SES (3/6). | "*At least for me and what I need to know and the way I function, make decisions, this is very useful. It tells me exactly what the kernel of what I need to know without overloading me with a lot of detail that to my mind muddies the waters or takes away from focusing on what is the critical information.*" - Patient, OG, Higher SES |
| Patients of higher SES who received the Option Grid were more likely to mention that the tool helped them compare their options (11/12) than patients of lower SES (4/6). | "*People are always going to want to know what the difference is between a lumpectomy and mastectomy in terms of outcomes and what you can expect. I think that’s the purpose of that tool, it’s just to line-up those options in a way that you can go back to it. Because you’re not going to remember everything that’s said to you but you can go back and say, 'How are they different? How are they the same? How do I make a decision?'*" - Patient, OG, Higher SES  "*It really helped me to decide on different things and it just helped me decide on what was good for me and what was coming. Sometimes when you first hear about this, you don’t know exactly what you’re in for or what it’s going to look like so it helped me to go step by step and not to go – it answered all of my questions, really.*" - Patient, OG, Higher SES |
| Patients of higher SES who received the Option Grid were more likely to use the tool at home for reassurance (5/12) compared to patients of lower SES (1/6). | “*It was to just go back all over this. I’d already made up my decision, so I went over it for reinforcement.*” - Patient, OG, Higher SES |
| *No differences* | |
| All patients of lower SES who received the Option Grid (6/6) and most patients of higher SES who received the Option Grid (11/12) felt that the tool was easier to understand than materials they have received before. | "*I just think this was more to the point. I have received a breast cancer book and that was great but it’s so entailed, and I found out that I had to put down after a while because I’m just looking up everything, things that probably didn’t even pertain to me, so this was more to the point.*" - Patient, OG, Higher SES |
| Patients of higher SES (12/12) and lower SES (6/6) who received the Option Grid all mentioned that the tool was easy to understand. All patients of higher SES who received the Option Grid mentioned that the tool was easy to use (12/12) compared to patients of lower SES who received the Option Grid (4/6). | “*It was really easy to use.*” - Patient, OG, Higher SES |
| **Cognitive participation - Who does the work?** | |
| *Differences* | |
| All patients of higher SES who received the Option Grid mentioned that they would prefer to receive a paper version (12/12) compared to patients of lower SES (1/6). | "*This is a very good paper. I like this paper. It’s kind of a big paper. At the end, it was easy to hold. It’s waxy, very nice.*" - Patient, OG, Higher SES |
| All patients of higher SES who received the Option Grid mentioned that their preference is to use the Option Grid with their provider (12/12) compared to patients of lower SES (4/6). | "*My personal opinion is that it should be used and handed to you by the surgeon and be used as a conversation tool.*" - Patient, OG, Higher SES  "*I understood it but she went more in-depth into it like stuff that’s not on the paper, she actually went more in-depth in it. I think when she brings it to you in a doctor visit, you can understand it to a better height.*" - Patient, OG, Higher SES |
| *No differences* | |
| All patients of higher SES (12/12) and lower SES (6/6) who received the Option Grid recommend that other patients like them should use the tool. | "*If I have a friend right now that’s making cancer decisions, I would show her the tool*." - Patient, OG, Higher SES  "*I would recommend it to everybody*." - Patient, OG, Lower SES |
| **Collective action - How does the work get done?** | |
| *Differences* | |
| All patients of lower SES who received the Option Grid (6/6) felt that their surgeon was comfortable using the tool compared to patients of higher SES (8/12). | "*He just was very confident. It wasn’t like he was bringing it out and saying, 'Okay, I have to go through this. I have to use this tool.' I didn’t sense that at all.*" - Patient, OG, Lower SES |
| patients of higher SES who received the Option Grid were more likely to mention that the tool was used with them at the right time (11/12) compared to patients of lower SES (4/6). | "*I think that was a good time to get the option grid so that we could look at it while we were discussing the surgical options. It wouldn’t have meant much if she had given it to me before [discussing pathology].*" - Patient, OG, Higher SES |
| *No differences* | |
| Both patients of higher SES (12/12) and lower SES (6/6) equally felt that the use of Option Grid did not feel awkward. | "*No, nothing was awkward at all.*" - Patient, OG, Lower SES |
| Patients of higher SES (8/12) and lower SES (4/6) who received the Option Grid equally felt that the conversation aids were a part of their surgeon's normal routine. | “*Like every patient* [the surgeon] *went through the tool with them? Yes.*” - Patient, OG, Higher SES |
| Patients of higher SES (10/12) and lower SES (5/6) who received Option Grid equally took their tool home with them after their appointment with their surgeon. | "*Yes. I took everything home*." - Patient, OG, Higher SES |
| **Reflexive monitoring - How is the work understood?** | |
| *Differences* | |
| Over half of patients of higher SES who received the Option Grid felt that the tool influenced their discussion with their surgeon (7/12) compared to patients of lower SES (1/6). | "*I think it really influenced our discussion by keeping us on track and talking about the risks/benefits of both options.*" - Patient, OG, Higher SES |
| However, patients of lower SES who received the Option Grid were more likely to mention that the tool affected their treatment decision (5/6) than patients of higher SES (6/12). | "*I’d say it really influenced my decision.*" - Patient, OG, Lower SES |
| Less than half of patients of higher SES (2/6) who received the Option Grid mentioned that the tool helped them understand their options, while half of patients of lower SES who received the Option Grid said the same (3/6). | "*It helped me to understand my options about what I wanted to do.*" - Patient, OG, Lower SES |
