# Supplementary material for: Implementation and sustainability factors of two early-stage breast cancer conversation aids in diverse practices
Source: Implement Sci. 2021 May 10;16:51. doi: 10.1186/s13012-021-01115-1 (PMC8108365; doi:10.1186/s13012-021-01115-1)
Supplement: Supplementary file 13 — Additional file 13. [file 13012_2021_1115_MOESM13_ESM.docx]

**Appendix 13. Differences between Option Grid and Picture Option Grid**

| **Construct themes** | **Quotations** |
| --- | --- |
| **Coherence - What is the work?** | |
| ***Patient*** | |
| Patients who received Option Grid were more likely to mention that the tool was concise (12/18) compared to patients who received Picture Option Grid (8/24). | "*It was more concise. Other materials I received, you had to read through a whole bunch of things before you could get to the end and decide."* - Patient, OG, Lower SES |
| ***Surgeon*** | |
| Two surgeons who used Picture Option Grid noticed that the inclusion of a question about the comparison of cost between the two treatments differed from their usual practice. | "*The only thing that’s different on that sheet was financial, truthfully, we don’t usually bring that up with patients unless they ask.*" - Surgeon, POG |
| All surgeons who used Option Grid (5/5) mentioned that the use of the conversation aids did not differ from their usual practice compared to only half of surgeons who used Picture Option Grid (3/6). | "*It wasn’t that different from what I do when I’m discussing the surgical options to a patient. From that end, it wasn’t anything different.*" - Surgeon, OG  "*I feel like the information was similar to my usual practice but definitely it was delivered in a little different way because normally I would just write out some of the things that are on the Picture Option Grid but I wouldn’t do it in such detail.*" - Surgeon, POG |
| Surgeons who used Option Grid (4/5) were much more likely to mention that the conversation aids were meant to help compare options compared to surgeons who used Picture Option Grid (1/6). | "*I liked that it was side by side so that the patient can easily compare the difference between the two.*" - Surgeon, OG |
| **Cognitive participation - Who does the work?** | |
| ***Patient*** | |
| Patients who received Picture Option Grid were more likely to recommend receiving the tool ahead of their appointment with their surgeon (12/24) compared to patients who received Option Grid (3/18). | "*I think if it had been emailed to me before, maybe it might have helped me, to have it before my office visit, process the information.*" - Patient, POG, Higher SES |
| More patients who received Option Grid indicated that receiving the tool from their surgeon is best (14/18) compared to patients who received Picture Option Grid who said the same (15/24). | “*Probably as the doctor is telling them the news. I think the doctor giving it to them after they’re given their diagnosis. It’s usually at that same visit, anyways.*” - Patient, OG, Higher SES |
| Almost all patients who received Option Grid indicated that paper-based versions of the tool are important (15/18) compared to patients who received Picture Option Grid (14/24). | "*Being someone who does not have any access to computers and all the rest, it’s much easier for me to have a hard copy. I can take it home with me*." - Patient, OG, Lower SES |
| ***Surgeon*** | |
| Surgeons who used Picture Option Grid (6/6) were much more likely to mention that they should give the tool at the surgical consultation compared to surgeons who used Option Grid (1/5). | “*It’s still something very good for actual surgeons to have and use during their conversations* [with patients].” - Surgeon, POG |
| Surgeons who used Picture Option Grid (4/6) were much more likely to mention, not prompted, being comfortable with a nurse giving the tool compared to surgeons who used Option Grid (0/5). | "*I think it’s great if your office has a navigator or someone who’s still going to follow up with the patient to potentially go through it with the patient, but it’s still something very good for actual surgeons to have and use during their conversations.*" - Surgeon, POG |
| **Collective action - How does the work get done?** | |
| ***Patient*** | |
| Almost all patients who received Option Grid (16/18) but over half of patients who received Picture Option Grid (16/24) mentioned that using the tool did not feel awkward. | "*No. Not [awkward] at all.*" - Patient, OG, Higher SES |
| ***Surgeon*** | |
| Surgeons who used Picture Option Grid (5/6) were more likely to mention that using the tool for the first few times was awkward compared to surgeons who used Option Grid (2/5). | "*Well, it became pretty much second-hand for me actually. I got really used to using it...It just became part of my routine.*" - Surgeon, POG |
| **Reflexive monitoring - How is the work understood?** | |
| ***Surgeon*** | |
| Surgeons who used Picture Option Grid (3/6) were more likely to mention that their colleagues liked the tool, not prompted, compared to surgeons who used Option Grid. (1/5). | "*My fellows, my surgical fellows, seem to like it as well. It kind of organizes their approach also when they were kind of thinking about how they would present patients in the future.*" - Surgeon, POG |
| Surgeons who used Option Grid (4/5) were more likely to write or draw on the tool compared to surgeons who used Picture Option Grid (2/6). | "*I would sometimes add notes and offer the patient an opportunity to ask questions.*" - Surgeon, OG  "*I think a lot of times I might circle things or put a check next to important things based on what the patient’s values or priorities might be based on our discussion and where things are going, so I might highlight that. ...I don’t think there was a whole lot of extra room for pictures.*" - Surgeon, OG |
| **Surgeon -** No differences | |
| All intervention surgeons mentioned that the time it took to use the tool didn't change the typical time they spend with patients in the surgical consultation (11/11). | "*I’m, again, using it as I got faster as I got more used to it. It did help the rhythm. At first, it took me a little bit longer than I think it would’ve been, but not much. Then at the end, I think it ultimately helped structure things. It might have made things as efficient or more efficient.*" - Surgeon, OG  "*It takes no more time to use the tool than it would not use the tool. It doesn’t add any extra time.*" - Surgeon, POG |
